# Use of stimulant drugs among high school adolescents in a Metropolitan Region: a school-based study^1^

**DOI:** 10.1590/1518-8345.8157.4964

**Published:** 2026-11-02

**Authors:** Franciéle Marabotti Costa Leite, Tiffani Matos Oliveira, Letícia Peisino Buleriano, Micael Franco Alves, Mayara Alves Luis, Carlos Augusto Lopes, Fernanda Garcia Gabira Miguez

**Affiliations:** 1 Universidade Federal do Espirito Santo, Vitória, ES, Brazil.; 2 Prefeitura de Vitória, Secretaria Municipal de Saúde de Vitória, Vitória, ES, Brazil.

**Keywords:** Adolescent, Central Nervous System Stimulants, Prevalence, Risk Factors, Cross-Sectional Studies, Sexual and Gender Minorities.

## Abstract

**Objective:**

to identify the prevalence of stimulant drugs among adolescents in high school and the associated factors.

**Method:**

cross-sectional study with 4,614 adolescents between 14 and 19 years old. Descriptive, bivariate (chi-square test and Fisher’s exact test) and multivariate (Poisson regression with robust variance) analyses were performed, adopting a significance level of p<0.05.

**Results:**

the prevalence of lifetime use was 6% (95% confidence interval: 5.4-6.8), and current use was 1.6% (95% confidence interval: 1.3-2.0). Higher prevalence of stimulant drug experimentation among adolescents aged 18 to 19 years (PR: 2.24), LGBTQIA+ (PR: 1.35), black race (PR: 1.55), with work (PR: 1.68) and whose parents live separately (PR: 1.62). Adolescents who belong to the Catholic or Evangelical religion had lower frequencies of experimentation and current use (p<0.05). Current use was more frequent among those of black race (PR: 2.94), class B (PR: 2.27), and those who have a job (PR: 2.69).

**Conclusion:**

it is observed that experimentation and use of stimulant drugs are present among adolescents and that sociodemographic and behavioral factors are associated with this practice. Integrated strategies between the health, education, social assistance and public security sectors are necessary for the prevention and mitigation of the problem in school, family and community environments.

## Highlights

**(1)**The study investigated stimulant drug use among high school adolescents.

**(2)**The prevalence of lifetime use was 6% and current use was 1.6%.

**(3)**Older age, work, black race-color, and being LGBTQIA+ increased the prevalence.

**(4)**Catholic or evangelical religion showed a protective effect against the use of stimulants.

**(5)**The findings support intersectoral prevention actions in the school and community context.

## Introduction

The World Health Organization (WHO) defines drugs as any substance not produced by the body that, when introduced into the body, can act on one or more systems, causing changes in their functioning^1^. In this context, psychoactive drugs are characterized by their ability to affect the mental and cognitive processes of individuals, influencing perceptions, emotions, behavior and consciousness^2^.

Historically, human beings have continued to be interested in psychoactive substances, and the first contact has occurred at an increasingly early age, such as in adolescence, which is a period of great vulnerability, as it is a time of search for identity, greater need for social acceptance and a tendency to test limits^3^ ^-^ ^5^.

In addition, brain immaturity, especially in regions related to self-control and decision-making, also contributes to impulsivity, which can increase vulnerability to risky behaviors. This scenario represents a growing concern in the context of public health, as it directly impacts the physical, emotional and social health of adolescents^6^.

Regarding the estimates, international evidence points to relevant prevalence of stimulant drug use among adolescents. A study conducted with 33,184 adolescents in five Southeast Asian countries identified that the prevalence of amphetamine use throughout life ranged from 1.2% in Laos to 4.8% in the Philippines and 5.1% in Timor-Leste^7^. In the United States, data from the Youth Risk Behavior Survey (YRBS) indicated that 5.7% of the adolescents interviewed reported cocaine use in 2015^8^. In Europe, it is observed that 2% of adolescents had tried cocaine at least once^9^. In Australia, a study indicates that 7.3% of individuals aged between 14 and 24 years reported cocaine use, while 7.6% indicated that they had used ecstasy in the 12 months prior to the survey^10^. 

In Brazil, a study carried out with 271 students in the interior of Pernambuco showed that 19.6% of the students reported having used some psychoactive substance in their lives and/or in the thirty days prior to the survey^5^. The II National Survey on drug use by the Brazilian population (2019) indicated that 2.5% of adolescents between 13 and 15 years old reported cocaine consumption^11^. On the other hand, the PeNSE survey (2019), conducted by the Ministry of Health, indicated that 1.2% of adolescents between 13 and 17 years of age had already tried this drug^12^, while 1.6% reported ecstasy use, with a slightly lower prevalence of crack and methamphetamine consumption^12^. These findings corroborate the estimates of the United Nations Office on Drugs and Crime (UNODC), which point to a progressive increase in the use of these drugs, especially in large urban centers.

In addition, the consumption of psychoactive drugs generates a heightened sense of well-being, euphoria, excitement and alertness, and increases in motor activity. However, they also reduce appetite and can result in insomnia, cardiovascular changes, greater exposure to accidents and violence^13^ ^-^ ^15^. In addition, consumption may be associated with low school performance, absenteeism and school dropout, and greater involvement in infraction behaviors and situations of social vulnerability, with possible repercussions throughout adult life. At high doses, they can lead to restlessness, agitation, altered perceptions of reality, and hallucinations. In addition, the effects of chronic use and withdrawal of these substances may include paranoia, psychosis, depression and/or suicidal ideation^14^ ^-^ ^17^. In family relationships, it has negative repercussions, promoting family conflicts and weakening of support bonds^14^ ^-^ ^16^.

In view of the above, the use of stimulant drugs among adolescents is an important public health problem and, in this sense, health professionals, especially nurses who work at different levels of health care, play a strategic role in prevention, promotion and health education actions in the school, family and community environments. However, despite the relevance of the theme, there are many gaps in the scientific literature, especially regarding regional prevalences and groups of greater vulnerability, a fact that justifies the present study, which aims to identify the prevalence of stimulant drugs among adolescents in high school and the associated factors.

## Method

### Study design

This is a cross-sectional epidemiological study carried out in the Metropolitan Region of Greater Vitória (RMGV) between March and December 2023, in public and private high schools.

### Setting

The study took place in the RMGV, consisting of: Cariacica, Fundão, Guarapari, Serra, Viana, Vila Velha and Vitória. The RMGV, according to the Brazilian Institute of Geography and Statistics (IBGE, 2020), has a population of approximately 1,880,828 people and contains an area of 281,760.93 km^2^. 

### Period

Data collection took place between March and December 2023.

### Population

The study population was composed of high school adolescents, aged 14 to 19 years, regularly enrolled in 63 public or private schools in the RMGV. The parents or guardians signed the Informed Consent Form (ICF) and the student the Informed Assent Form (TALE). As an exclusion criterion, the lack of understanding due to the participant’s intellectual deficit, so that there would be no intellectual conditions for the student to respond to the instrument.

### Sample definition

To calculate the sample, aiming at representativeness of the public and private networks, the sampling was by cluster and stratification. Thus, for each stratum, simple samples were performed, being corrected for complex samples in a second moment using SPSS 26, ensuring 95% confidence and a maximum error of 5%. In addition, for each stratum of municipality, the sample parameters were 95% confidence and a maximum error of 10%.

It is worth noting that the training of the research team and the pilot test were carried out, which took place in February 2023, in which 50 adolescents who did not make up the final sample of the study participated. The pilot test was important for making small adjustments regarding sociodemographic and behavioral issues, in order to promote greater clarity of the questions, as well as being fundamental for estimating the response time to the questionnaire, which was on average from forty to fifty minutes. Data collection took place on tablets using REDCAP (Research Electronic Data Capture), with socioeconomic (gender, age, race, income, family situation) and behavioral (use of cocaine, crack, ecstasy and methamphetamine) issues.

### Study variables

The outcome variable was composed of the use of stimulant drugs according to the adolescents’ self-report, the answers were dichotomized into yes and no, and the use of stimulant drugs was considered the adolescent who in at least one of the items presented a positive report of the use of any of the following drugs: cocaine, crack, ecstasy and/or methamphetamine at least once in life and current use. 

The independent variables were: sex (female or male), age group (14 to 15 years, 16 to 17 years, 18 to 19 years), gender identity (cisgender or non-cisgender), sexual orientation (heterosexual or LGBTQIA+), race-color (white, black and brown), in a relationship (yes or no), has a job, job or business (yes or no), economic classification (A; B; C/D/E), parents live together (yes or no), type of school (public or private); religion (none; Catholic; evangelical; other).

### Data processing and analysis

Statistical analysis was initially conducted by describing the data through the presentation of absolute and relative frequencies and 95% confidence intervals (95%CI), and exclusion was considered per complete case, followed by bivariate analysis using Pearson’s Chi-square test (χ²) and Fisher›s exact test, to verify the possible relationships between the variables. Subsequently, crude and adjusted multivariate analysis of the Prevalence Ratio (PR) was performed by Poisson regression with robust variance, and variables with statistical significance of 0.20 were considered for entry into the adjustment, and those with a value of p<0.05 were maintained. All analyses were performed using the Stata 17.0 statistical program. 

### Ethical aspects

The project was submitted to the Research Ethics Committee of the Federal University of Espírito Santo, and was approved by opinion No. 5,900,370, in order to comply with Resolution No. 466/2012. And it obtained the signature of those responsible for the Informed Consent Form (ICF) and the adolescents of the Informed Assent Form (TALE). 

## Results

The prevalence of ever lifetime use of a stimulant drug among schoolchildren was 6% (95%CI 5.4-6.8), and currently 1.6% use some stimulant drug (95%CI 1.3-2.0). The prevalence of schoolchildren who reported having used cocaine at least once in their lives was 1.2% (95%CI 1.0-1.6), and the current use of this drug is 0.2% (95%CI 0.1-0.4). Regarding the use of crack or *merla*, the prevalence was 0.1% (N=6, 95%CI 0.0-0.2), and the current use was 0.1% (95%CI 0.0-0.2). Approximately 5% of the sample stated that they had experienced ecstasy or bullet or MD in their lifetime, and currently 1.4% use this drug (95%CI 1.1-1.8). Regarding methamphetamine use, 0.6% of the interviewees stated that they had already tried methamphetamine in their lives (95%CI 0.4-0.9) and 0.1% currently use methamphetamine (95%CI 0.0-0.3) (Table 1).

**Table 1:** Use of stimulant drugs among high school students. Metropolitan Region of Greater Vitória, ES, Brazil, March to December, 2023

	N*	%^†^	95%CI^‡^
Use once in a lifetime			
**Stimulant drugs**			
No	4334	94.0	93.2-94.6
Yes	278	6.0	5.4-6.8
**Cocaine**			
No	4555	98.8	98.4-99.0
Yes	57	1.2	0.9-1.6
**Crack or *merla***			
No	4604	99.9	99.7-99.9
Yes	06	0.1	0.01-0.3
**Ecstasy**			
No	4383	95.1	94.4-95.7
Yes	227	4.9	4.3-5.6
**Methamphetamine**			
No	4581	99.4	99.1-99.6
Yes	29	0.6	0.4-0.9
**Currently use**			
**Stimulant drugs**			
No	4538	98.4	97.9-98.7
Yes	74	1.6	1.3-2.0
**Cocaine**			
No	4602	99.8	99.6-99.9
Yes	10	0.2	0.1-0.4
**Crack or *merla***
No	4607	99.9	99.8-100.0
Yes	3	0.1	0.0-0.2
**Ecstasy**			
No	4544	98.6	98.2-98.9
Yes	66	1.4	1.1-1.8
**Methamphetamine**
No	4603	99.9	99.7-99.9
Yes	7	0.1	0.0-0.3

*N = Absolute frequency; ^†^% = Prevalence; ^‡^95%CI = 95% Confidence interval

According to Table 2, the age at which stimulant drugs were used for the first time was 15 years or older for all substances analyzed, and the proportion of initiation of use from this age was 50% for cocaine (95%CI 36.7-63.3), 50% for crack (95%CI 10.9-89.1), 77.9% for ecstasy (95%CI 72.0-82.8) and 64% for methamphetamine use (95%CI 42.9-80.8). Also, according to the participants, 32.1% obtained cocaine from a friend (95%CI 21.1-45.7), and, likewise, ecstasy was acquired through friends for about 39% of the adolescents (95%CI 33.0-45.7). On the other hand, crack/*merla* had a higher frequency of purchase in stores, bars, pubs, with a salesperson and/or in the club (50%; 95%CI 10.9-89.1), and, finally, about 28% of the adolescents bought and/or nightclubs (“*baladas*”) (13.8%, 95%CI 5.0-32.5) or got methamphetamine with friends (13.8%, 95%CI 5.0-32.5). It is worth noting the high prevalence of adolescents who report not remembering how the substance was acquired for all drugs.

**Table 2:** Use of stimulant drugs among high school students. Metropolitan Region of Greater Vitória, ES, Brazil, March to December, 2023

	Cocaine			**Crack/*merla***			Ecstasy/bullet/MD			Methamphetamine		
	N*	%^†^	95%CI ^‡^	N*	% ^†^	95%CI^‡^	N*	%^†^	95%CI^‡^	N*	%^†^	95%CI^‡^
How old were you when you first tried it?												
Less than 10 years	10	18.5	10.1-31.5	2	33.3	5.1-82.2	7	3.1	1.5-6.4	3	12.0	3.7-32.7
11 to 14 years	17	30.5	20.3-45.3	1	16.7	1.2-77.0	43	19.0	14.4-24.7	6	24.0	10.7-45.4
15 or more	27	50.0	36.7-63.3	3	50.0	10.9-89.1	176	77.9	72.0-82.8	16	64.0	42.9-80.8
**How did you do it?**												
I bought it in a store, bar, bar, with a salesman and/or in the club (“*balada*”)	9	16.1	8.5-28.4	3	50.0	10.9-89.1	54	23.8	18.7-29.8	4	13.8	5.0-32.5
I gave money to someone to buy for me	4	7.1	2.6-17.9	1	16.7*	1.2-77.0	9	4.0	2.1-7.5	-	-	-
I caught it hidden at home	3	5.4	1.7-15.7	-	-	-	-	-	-	2	6.9	1.2-25.0
An older person gave me	1	1.8	2.4-12.1	-	-	-	21	9.3	6.1-13.8	-	-	-
Someone in my family gave it to me	1	1.8	2.4-12.1	1	16.7*	1.2-77.0	4	1.8	0.7-4.2	3	10.3	3.2-28.7
I got it with a friend	18	32.1	21.1-45.7	-	-	-	89	39.2	33.0-45.7	4	13.8	5.0-32.5
I got it at school	-	-	-	-	-	-	-	-	-	1	3.5	0.4-22.3
I don’t remember and another way	20	35.7	24.1-49.3	1	16.7*	14.3	50	22.0	17.1-27.9	15	51.7	33.4-69.6

Note: no possibility to round, percentage above; *N = Absolute frequency; ^†^% = Prevalence; ^‡^95%CI = 95% confidence interval

Table 3 shows the distribution of stimulant drug use among high school students, according to the characteristics under study. A relationship is noted between experimentation and the use of stimulants and the variables: gender identity, sexual orientation, race/skin color, marital status, type of school, current work and religion (p<0.05). On the other hand, the variables age and marital status of the parents only showed a significant relationship with the experimentation of stimulants (p<0.001), and the current use of the drug with gender and economic class (p<0.05).

**Table 3:** Distribution of stimulant drug use among high school students in the Metropolitan Region of Greater Vitória. ES, Brazil, March to December, 2023

Variables	Stimulant experimentation				Current use of stimulants			
	N*	%^†^	95%CI^‡^	p-value	N*	%^†^	95%CI^‡^	p-value
**Gender^§^**				0.137				0.009
Female	139	5.5	4.72-6.52		29	1.2	0.81-1.66	
Male	139	6.6	5.61-7.74		45	2.1	1.60-2.84	
**Age (years)**				<0.001				0.248
14 to 15	37	3.8	2.3-5.2		12	1.2	0.70-2.27	
16 to 17	179	5.9	5.1-6,8		48	1.6	1.29-2.09	
18 to 19	62	10.3	8.0-13.0		14	2.3	1.38-3.88	
**Gender identity**				0.032				0.010
Cisgender	254	5.8	5.18-6.58		64	1.5	1.16-1.89	
Non-cisgender	26	8.9	6.15-12.8		10	3.4	1.86-6.27	
**Sexual orientation**				<0.001				0.049
Heterosexual	193	5.3	4.59-6.04		52	1.4	1.10-1.86	
LGBTQIA+	85	9.0	7.31-11.00		22	2.3	1.53-3.50	
**Race/Skin color**				0.001				0.003*
White	83	4.7	3.8-5.8		16	0.9	0.60-1.48	
Black	66	8.7	6.9-10.9		21	2.8	1.81-4.20	
Brown	113	5.8	4.8-6.3		30	1.5	1.08-2.19	
**Marital status**				0.001				0.006
With partner	98	8.0	6.59-9.63		44	1.3	0.97-1.74	
No companion	180	5.3	4.61-6.12		30	2.4	1.71-3.47	
**Socioeconomic classification**				0.957				0.017*
A	497	6.2	4.89-8.40		10	1.3	0.71-2.42	
B	129	5.9	5.05-7.05		47	2.2	1.62-2.86	
C//D/E	102	6.1	5.12-7.44		17	1.0	0.63-1.63	
**Type of school**				<0.001				0.019
Public	217	7.0	6.17-8.00		59	1.9	1.48-2.46	
Private	61	4.0	3.13-5.12		15	1.0	0.59-1.63	
**Currently, he has a work, job, or business**				<0.001				<0.001
No	176	5.04	4.36-5.81		40	1.1	0.84-1.56	
Yes	102	9.12	7.58-11.00		31	3.0	2.18-4.23	
**Religion**				<0.001				<0.001
None	91	8.4	6.85-10.15		25	2.3	1.56-3.38	
Catholic	55	4.7	3.60-6.33		11	0.9	0.52-1.68	
Evangelical	89	4.4	3.62-4.43		22	1.1	0.72-1.66	
Other	43	12.7	9.57-16.72		16	4.7	2.92-7.59	
**Marital status of parents - they live together**				<0.001				0.056
Yes	99	4.10	3.37-4.96		29	1.2	0.83-1.72	
No	174	8.04	7.00-9.26		41	1.9	1.40-2.56	

*N = Absolute frequency; ^†^% = Prevalence; ^‡^95%CI = 95% Confidence Interval; ^§^Fisher´s exact

After adjusting for confounding factors, it was observed that those aged 18 to 19 years had a 2.24 times higher prevalence (95%CI 1.47-3.40) of stimulant drug experimentation compared to adolescents aged 14 to 15 years. Self-identified LGBTQIA+ schoolchildren had a 35% higher prevalence of experimentation (95%CI 1.04-1.77) compared to heterosexuals, as well as black students had a 1.55-fold higher prevalence of experimentation with stimulant drugs (95%CI 1.13-2.14) compared to white adolescents. Experimentation with stimulants at some time in their lifetime was 68% more prevalent among students with work (95%CI 1.32-2.13). Belonging to the Catholic religion reduced the prevalence of stimulant drug experimentation by 30% (PR: 0.70; 95%CI 0.50-0.99), as well as in evangelical religion, with a reduction of 41% (PR: 0.59; 95%CI 0.44-0.81) compared to adolescents who reported having no religion. Finally, adolescents whose parents live apart had a 62% higher frequency of experimentation with stimulant drugs (95%CI 1.26-2.10) compared to those whose parents live together (Table 4).

**Table 4:** Crude and adjusted prevalence ratio of stimulant drug experimentation among high school students. Metropolitan Region of Greater Vitória, ES, Brazil, March to December, 2023

Variables	Stimulant Drug Experimentation					
	Crude PR*	95%CI^†^	p-value	Adjusted PR^‡^	95%CI^†^	p-value
**Gender**			0.138			0.155
Female	1.0			1.0		
Male	1.19	0.95-1.49		1.20	0.93-1.53	
**Age (years)**			<0.001			<0.001
14 to 15	1.0			1.0		
16 to 17	1.55	1.09-2.19		1.48	1.03-2.13	
18 to 19	2.69	1.81-3.99		2.24	1.47-3.40	
**Gender identity**			0.031			0.862
Cisgender	1.0			1.0		
Non- cisgender	1.53	1.04-2.25		0.96	0.61-1.51	
**Sexual orientation**			<0.001			0.027
Heterosexual	1.0			1.0		
LGBTQIA+	1.70	1.33-2.17		1.35	1.04-1.77	
**Race-skin color**			<0.001			0.025
White	1,0			1.0		
Black	1.86	1.36-2.54		1.55	1.13-2.14	
Brown	1.24	0.94-1,63		1.19	0.90-1.58	
**Marital status**			0.001			0.063
With partner	1.50	1.18-1.90		1.27	0.99-1.63	
No companion	1.0			1.0		
**Socioeconomic classification**			0.957			-
A	1.01			-	-	
B	0.98	0.75-1.25		-	-	
C//D/E	1.0	0.72-1.41		-	-	
**Type of school**			<0.001			0.177
Public	1.75	1.33-3.31		1.24	0.91-1.68	
Private	1.0			1.0		
**Currently, he has a work, job, or business**			<0.001			<0.001
No	1.0			1.0		
Yes	1.81	1.43-2.29		1.68	1.32-2.13	
**Religion**			<0.001			<0.001
None	1.0			1.0		
Catholic	0.56	0.40-0.77		0.70	0.50-0.99	
Evangelical	0.53	0.40-0.70		0.59	0.44-0.81	
Other	1.52	1.08-2.14		1.36	0.94-1.95	
**Marital status of parents - they live together**			<0.001			<0.001
Yes	1.0			1.0		
No	1.96	1.53-2.51		1.62	1.26-2.10	

*Crude PR = Crude prevalence ratio; ^†^95%CI = 95% confidence interval; ^‡^Adjusted PR = Adjusted prevalence ratio

The current use of stimulant drugs after adjusting the data was more prevalent for black adolescents (PR 2.94; 95%CI 1.53-5.65) compared to white adolescents. Compared to adolescents belonging to lower economic classifications (C/D/E), adolescents belonging to class B had a prevalence 2.27 times higher in the current use of stimulant drugs (95%CI 1.31-3.95). Having a job led to a 2.69-fold higher prevalence of current use (95%CI 1.69-4.30) compared to adolescents without work, and the prevalence of current stimulant drug use was 58% lower in those belonging to the Catholic religion (95%CI 0.20-0.87) and 57% lower in evangelical schoolchildren (95%CI 0.24-0.78), compared to adolescents who reported having no religion (Table 5).

**Table 5:** Crude and adjusted analysis of current stimulant drug use among high school students. Metropolitan Region of Greater Vitória, ES, Brazil, March to December, 2023

Variables	Current use of stimulant drugs					
	Crude PR*	95%CI^†^	p-value	Adjusted PR^‡^	95%CI^†^	p-value
**Gender**			0.010			0.103
Female	1.0			1.0		
Male	1.84	1.16-2.93		1.49	0.92-2.41	
**Age (years)**			0.254			-
14 to 15	1.0			-	-	
16 to 17	1.28	0.68-2.40		-	-	
18 to 19	1.87	0.87-4.02		-	-	
**Gender identity**			0.012			0.445
Cisgender	1.0			1.0		
Non-cisgender	2.32	1.20-4.47		1.39	0.59-3.28	
**Sexual orientation**			0.050			0.223
Heterosexual	1.0			1.0		
Non-heterosexual	1.63	1.00-2.68		1.45	0.80-2.64	
**Race/Skin color**			0.003			0.005
White	1.0			1.0		
Black	3.06	1.61-5.84		2.94		
Brown	1.71	0.93-3.12		1.74	1.53-5.65	
**Marital status**			0.007		0.97-3.12	0.098
With partner	1.0			1.0		
No companion	1.89	1.19-2.97		1.52	0.93-2.49	
**Socioeconomic classification**			0.019			0.009
A	2.12	0.59-2.79		1.26	0.52-3.03	
B	1.29	1.22-3.67		2.27	1.31-3.95	
C//D/E	1.0			1.0		
**Type of school**			0.021			0.672
Public	1.94	1.10-3.40		1.14	0.61-2.13	
Private	1.0			1.0		
**Currently, he has a work, job or business**			<0.001			<0.001
No	1.0			1.0		
Yes	2.66	1.69-4.18		2.69	1.69-4.30	
**Religion**			<0.001			<0.001
None	1.0			1.0		
Catholic	0.41	0.20-0.82		0.42	0.20-0.87	
Evangelical	0.48	0.27-0.84		0.43	0.24-0.78	
Spiritist	2.06	1.11-3.82		1.49	0.75-2.94	
**Marital status of parents- do they live together?**			0.060			0.395
Yes	1.0			1.0		
No	1.58	0.98-2.54		1.24	0.76-2.03	

*Crude PR = Crude prevalence ratio; ^†^95%CI = 95% confidence interval; ^‡^Adjusted PR = Adjusted prevalence ratio

## Discussion

The prevalence of stimulant drug use at least once in life among the schoolchildren in this study was 6% (95%CI: 5.4-6.8), while current use was 1.6% (95%CI: 1.3-2.0). These findings are in line with data released by the North American study Monitoring the Future (MTF, 2022), which investigated high school adolescents up to 18 years of age, identifying a similar prevalence of use at least once in life (6.1%) and current use (1.5%) of stimulant drugs. In the same sense, a study carried out in Chile with adolescents aged 13-17 years also observed a prevalence of 5.9% of stimulant use at least once in life and 1.7% of current use^18^.

Regarding cocaine use, it was observed that the prevalence of experimentation in life among schoolchildren was 1.2% (95%CI: 1.0-1.6). This result is in line with a study carried out in Nigeria, in which 1.4% of adolescents reported using this substance throughout their lives^19^. Similarly, a study conducted in Macau, China, identified a prevalence of 1.2% of cocaine use at least once in life among adolescents^20^. On the other hand, a study carried out in Brazil, in the city of Cuiabá, found a higher prevalence of cocaine use throughout life (3.1%)^21^. Regarding current cocaine use, a prevalence of 0.2% (95%CI: 0.1-0.4) was observed. This result is similar to that described in studies with the adolescent population, in which 0.4% of the adolescents reported current cocaine use^19^.

Regarding the consumption of crack or *merla*, the prevalence found was 0.1% (95%CI 0.0-0.2). This result is lower than that identified by the European School Research Project on Alcohol and Other Drugs, which analyzed data from 99,647 adolescents in 35 countries and found an average prevalence of 1.1% of crack use at least once in life. In this study, the highest prevalence was observed in Cyprus (3.1%), while the lowest occurred in North Macedonia (0.2%) and Slovakia (0.5%)^9^. Similarly, a cross-sectional study carried out in Brazil, in the city of Cuiabá (MT), identified a prevalence of 1.4% of crack use at least once in life among adolescents^21^.

With regard to the use of ecstasy, candy or MD, the prevalence at some time in life was 5%, similar to that identified in the European School Survey Project on Alcohol and Other Drugs (ESPAD)^9^, which revealed a prevalence of 5.1% among high school adolescents in the Netherlands. Similarly, the prevalence of current use found (1.4%; 95%CI 1.1-1.8) is in line with that recorded in the ESPAD^9^, but with a focus on adolescents in the Czech Republic, where 1.7% reported consumption of these drugs.

The prevalence of methamphetamine use at least once in life among schoolchildren was 0.6% (95%CI: 0.4-0.9), a value higher, although not distant, than that observed in the study by the Brazilian Center for Information on Psychotropic Drugs (CEBRID, 2020), which identified a prevalence of less than 0.1% for the use of this substance. In addition, the current use of methamphetamine in this study was 0.1% (95%CI: 0.0-0.3), indicating low consumption among schoolchildren, a result similar to that described by ESPAD (2019), which pointed to a prevalence of 0.3% among adolescents^9^.

The analysis of the age at which stimulant drugs use begins reveals a worrying trend that, for all the substances analyzed, the first experience occurs from the age of 15. About 27.9% of individuals start using stimulant drugs before the age of 18, with a more pronounced trend among men, which may reflect social, cultural and biological differences in the propensity to use^15^. The early initiation of the use of these substances is closely associated with the development of more severe problems related to drug abuse and dependence in adulthood^16^.

The survey data indicate that the acquisition of stimulants occurs through friends or by shopping in stores, bars, bars, with a salesperson and/or at the club. Studies have reported a higher frequency of young people acquiring stimulant drugs (ecstasy, amphetamine, cocaine) in bars, nightclubs, parties, etc^22^ ^-^ ^25^. This is likely to occur because parties such as raves are more likely to be associated with higher chances of using these drugs, since drugs are used at such parties to improve music, lights, socialization and dancing all night^22^. 

Another finding was the higher prevalence of experimentation with stimulants among older adolescents aged 18 to 19 years (95%CI 1.47-3.40). As well as the findings in the present study, it was identified that the use of this type of drugs is done by older young people^14^. In addition, there are reports in the literature of a mean age of 18-19 years for cannabis and 21-24 years for cocaine^26^. Furthermore, between 2000 and 2001, there was already evidence of a large increase in the use of amphetamine-type stimulants among adolescents in Asia and Europe^27^. 

In addition, there was greater experimentation with stimulants among young people who declared themselves LGBTQIA+, with 35% more prevalence (95%CI 1.04-1.77). This data is similar to that of other studies, which suggest that this population group is at greater risk of using these substances related to minority stressors, such as the stigma suffered^28^ ^-^ ^29^.

In addition, in our study, there is a higher frequency of experimentation and current use of stimulants among black people, data that have not yet been explored in the literature. A study conducted in the United States shows that young people with white skin color are more likely to receive treatment, and have more access to private health services^30^.

The present study found a higher prevalence of experimentation and current use of stimulants among working students, data in line with a study that used data from more than 109 thousand Brazilian students and found a higher frequency of drug use among working adolescents, and suggests that paid work can enable greater financial independence and influence the autonomy of adolescents in decision-making for drug consumption^31^.

The association between the use of stimulants and social markers such as race/color and belonging to socially stigmatized groups, such as LGBTQIA+ people, highlights the role of structural inequalities in the production of health vulnerabilities. Structural racism and prejudice act as social determinants that increase exposure to chronic stressors, weaken support networks and restrict timely access to health and social protection services, especially during adolescence^32^. In this context, minority stress theory is an intersectional approach, according to which the overlapping of multiple minority markers intensifies exposure to risk factors and potentiates negative impacts on mental health. She maintains that persistent experiences of stigmatization, discrimination and violence increase the risk of psychological suffering and substance use as a coping strategy^33^.

Belonging to the Catholic or Evangelical religion decreased the prevalence of experimentation and current use of stimulant drugs compared to adolescents who declared having no religion. The findings corroborate a cross-sectional study carried out in the interior of São Paulo, in which students who practiced a religion were less likely to use drugs^34^. Religiosity arouses a mixture of fear and taboo, and, in the form of beliefs and practices, can be seen as a strong factor of resilience and resistance against the use of substances by adolescents^35^.

Another finding was the current use more frequent in adolescents who belong to higher economic classifications. A cross-sectional survey in Nigeria of more than 1,000 adolescents in public and private schools found that students who attend schools in areas populated by people with higher incomes were more likely to abuse drugs, including cocaine. It is possible that there is a relationship with the greater purchasing power available to students in these areas^19^. 

It was also found that adolescents whose parents live apart had a 62% higher frequency of experimentation with stimulant drugs (95%CI 1.26-2.10) compared to those whose parents live together. This result is in line with a study conducted in the United States with data from more than 41 thousand adolescents, in which those belonging to single-parent and non-parental families were more likely to use cocaine/heroin/methamphetamine (OR = 1.63-1.78, 95% CI = 1.21-2.71) than young people in two-parent families^36^. One of the possible justifications would be that families with two parents are more likely to monitor young people closely, so they would be less likely to be exposed to risky behaviors^36^. In addition, in addition to the parents’ relationship, the time dedicated to their children can influence the consumption of psychoactive substances^37^.

Considering the results presented and the impacts of the use of psychoactive substances on health, nursing professionals play an important role in this scenario. Nursing care for adolescents who use drugs or are at high risk of using drugs should be based on a welcoming attitude, without prejudice and capable of providing a space for dialogue. Health promotion and prevention strategies should be used, guided by the best scientific evidence, focusing on the subject’s autonomy and harm reduction^38^. The actions can be carried out in the individual nursing consultation itself or in collective activities. In this way, it is possible to outline strategies to make them more reflective, competent and confident. When potentialities are stimulated, self-esteem is strengthened, so as to enable conscious choices and decisions^39^. 

The cross-sectional design has limitations regarding the determination of causality, since the outcome is evaluated at the same time as the exposure, and this prevents the identification of the temporality of the events, requiring caution when evaluating the relationships between the variables explored. Also, information bias can also occur, compromising the accuracy of the answers obtained, however, it is worth considering that the students were able to answer the questionnaire individually, respecting their privacy, thus reducing the bias mentioned above. 

## Conclusion

We can conclude that 6% of high school adolescents have already used stimulant drugs at some point in their lives, and that 1.6% currently use them. The age of use for the first time, for most, was 15 years or older, and the acquisition of this substance happens mainly in stores, bars, bars, with a salesperson and/or in the club. Higher prevalence of experimentation was observed among older adolescents (18 to 19 years old) who self-declared themselves LGBTQIA+, black, schoolchildren with work and whose parents live separately. The current use is more frequent among those belonging to class B, of black race, and who work. Those who have a religion (evangelical or Catholic) had lower prevalence of experimentation and current use.

The findings of the present study reinforce the need for coping strategies against the use of stimulant drugs among adolescents in the community, family and school contexts. Efforts to coordinate intersectoral health, education, social assistance and public safety, as well as to include the family in this process, should be used to prevent and mitigate use. In the academic context, it is essential to carry out studies with a qualitative approach that explore, mainly, the reasons related to higher consumption among these adolescents, in addition to the development of research on interventions based on the objective of strengthening prevention actions. Finally, it is necessary to enhance the factors that reduce exposure to the risk of using these substances in order to promote healthy youth.

## Data Availability

Datasets related to this article will be available upon request to the corresponding author.
