# Supplementary material for: Use of stimulant drugs among high school adolescents in a Metropolitan Region: a school-based study
Source: Rev Lat Am Enfermagem. 2026 Nov 2;34:e4964. doi: 10.1590/1518-8345.8157.4964 (PMC13730520; doi:10.1590/1518-8345.8157.4964)
Supplement: Translation

# Translation: Uso de drogas estimulantes entre adolescentes do ensino médio de uma Região Metropolitana: um estudo de base escolar^*^

**DOI:** 10.1590/1518-8345.8157.4965

**Authors:** Franciéle Marabotti Costa Leite, Tiffani Matos Oliveira, Letícia Peisino Buleriano, Micael Franco Alves, Mayara Alves Luis, Carlos Augusto Lopes, Fernanda Garcia Gabira Miguez

**Affiliations:** 1 Universidade Federal do Espirito Santo, Vitória, ES, Brasil.; 2 Prefeitura de Vitória, Secretaria Municipal de Saúde de Vitória, Vitória, ES, Brasil.

**Keywords:** Adolescentes, Estimulantes do Sistema Nervoso Central, Prevalência, Fatores de Risco, Estudos Transversais, Minorias Sexuais e de Gênero.

## Abstract

**Objetivo:**

identificar a prevalência de drogas estimulantes entre adolescentes do Ensino Médio e os fatores associados.

**Método:**

estudo transversal com 4.614 adolescentes, entre 14 e 19 anos. Foram realizadas análises descritivas, bivariadas (teste do qui-quadrado e teste exato de Fisher) e multivariadas (regressão de Poisson com variância robusta), adotando-se nível de significância de p<0,05.

**Resultados:**

a prevalência de uso alguma vez na vida foi de 6% (intervalo de confiança de 95%: 5,4-6,8), e o uso atual foi de 1,6% (intervalo de confiança de 95%: 1,3-2,0). Maiores prevalências de experimentação de drogas estimulantes entre adolescentes de 18 a 19 anos (RP: 2,24), LGBTQIA+ (RP: 1,35), raça-cor preta (RP: 1,55), com trabalho (RP: 1,68) e cujos pais vivem separados (RP: 1,62). Adolescentes que pertencem à religião católica ou evangélica apresentaram menores frequências de experimentação e uso atual (p<0,05). O uso atual foi mais frequente entre os de raça-cor preta (RP 2,94), classe B (RP: 2,27), e, que possuem trabalho (RP: 2,69).

**Conclusão:**

observa-se que a experimentação e o uso de drogas estimulantes estão presentes entre os adolescentes e que fatores sociodemográficos e comportamentais estão associados essa prática. Estratégias integradas entre os setores da saúde, educação, assistência social e segurança pública são necessárias para a prevenção e mitigação do problema em ambientes escolares, familiares e comunitários.

## Destaques

**(1)** O estudo investigou o uso de drogas estimulantes entre adolescentes do Ensino Médio.

**(2)** A prevalência de uso alguma vez na vida foi de 6% e o uso atual de 1,6%.

**(3)** Idade mais elevada, trabalho, raça-cor preta e ser LGBTQIA+ aumentaram a prevalência.

**(4)** Religião católica ou evangélica mostrou efeito protetor contra o uso de estimulantes.

**(5)** Os achados subsidiam ações intersetoriais de prevenção no contexto escolar e comunitário.

## Introdução

A Organização Mundial da Saúde (OMS) define droga como qualquer substância não produzida pelo organismo que, ao ser introduzida no corpo, é capaz de atuar sobre um ou mais sistemas, provocando alterações em seu funcionamento^1^. Nesse contexto, as drogas psicoativas caracterizam-se pela capacidade de afetar os processos mentais e cognitivos dos indivíduos, influenciando percepções, emoções, comportamento e consciência^2^. 

Historicamente, observa-se um interesse contínuo do ser humano pelas substâncias psicoativas, e o primeiro contato tem acontecido de forma cada vez mais precoce, como por exemplo na adolescência, que constitui um período de grande vulnerabilidade, uma vez que é um momento de busca por identidade, maior necessidade de aceitação social e uma tendência a testar limites^3^ ^-^ ^5^.

Somando-se a isso, a imaturidade cerebral, especialmente nas regiões relacionadas ao autocontrole e à tomada de decisões, também contribui para a impulsividade, o que pode aumentar a vulnerabilidade a comportamentos de risco. Tal cenário representa uma preocupação crescente no contexto da saúde pública, uma vez que impacta diretamente na saúde física, emocional e social dos adolescentes^6^. 

No que tange às estimativas, evidências internacionais apontam prevalências relevantes do uso de drogas estimulantes entre adolescentes. Estudo realizado com 33.184 adolescentes em cinco países do Sudeste Asiático identificou que a prevalência do uso de anfetaminas ao longo da vida variou de 1,2% no Laos a 4,8% nas Filipinas e 5,1% em Timor-Leste^7^. Nos Estados Unidos, dados da Pesquisa do Comportamento de Risco do Jovem (YRBS), indicaram que 5,7% dos adolescentes entrevistados relataram uso de cocaína em 2015^8^. Na Europa, observa-se que 2% dos adolescentes haviam experimentado cocaína pelo menos uma vez^9^. Na Austrália, estudo indica que 7,3% dos indivíduos com idades entre 14 e 24 anos relataram o uso de cocaína, enquanto 7,6% indicaram ter usado ecstasy nos 12 meses anteriores à pesquisa^10^. 

No Brasil, pesquisa realizada com 271 estudantes no interior de Pernambuco mostrou que 19,6% dos estudantes relataram ter feito uso de alguma substância psicoativa na vida e/ou nos trinta dias que antecederam a pesquisa^5^. O II Levantamento Nacional sobre o uso de drogas pela população brasileira (2019) apontou que 2,5% dos adolescentes entre 13 e 15 anos relataram o consumo de cocaína^11^. Por outro lado, a pesquisa PeNSE (2019), realizada pelo Ministério da Saúde, indicou que 1,2% dos adolescentes entre 13 e 17 anos já haviam experimentado essa droga^12^, enquanto 1,6% relataram uso de *ecstasy*, com uma prevalência um pouco mais baixa de consumo de crack e metanfetamina^12^. Esses achados corroboram as estimativas do Escritório das Nações Unidas sobre Drogas e Crime (UNODC), que apontam para um aumento progressivo do uso dessas drogas, principalmente nos grandes centros urbanos. 

Ademais, o consumo das drogas psicoativas gera uma sensação elevada de bem-estar, euforia, excitação e alerta, e aumentos na atividade motora. No entanto, também reduzem o apetite e podem resultar em insônia, alterações cardiovasculares, maior exposição a acidentes e violências^13^ ^-^ ^15^. Além disso, o consumo pode estar associado ao baixo rendimento escolar, absenteísmo e evasão escolar, e maior envolvimento em comportamentos infracionais e situações de vulnerabilidade social, com possíveis repercussões ao longo da vida adulta. Em doses elevadas, podem levar à inquietação, agitação, percepções alteradas da realidade e alucinações. Ainda, os efeitos do uso crônico e da abstinência dessas substâncias podem incluir paranoia, psicose, depressão e/ou ideação suicida^14^ ^-^ ^17^. Em relações familiares, repercute negativamente, promovendo conflitos familiares e fragilização dos vínculos de apoio^14^ ^-^ ^16^.

Diante do exposto, constata-se o uso de drogas estimulantes entre adolescentes como um importante problema de saúde pública e, nesse sentido, os profissionais de saúde, em especial, o enfermeiro que atua em diferentes níveis de atenção à saúde, desempenham função estratégica nas ações de prevenção, promoção e educação em saúde no ambiente escolar, familiar e comunitário. Todavia, apesar da relevância da temática, nota-se na literatura científica muitas lacunas, em especial acerca das prevalências regionais e grupos de maior vulnerabilidade, fato que justifica a presente pesquisa que tem o objetivo de identificar a prevalência de drogas estimulantes entre adolescentes do Ensino Médio e os fatores associados. 

## Método

### Delineamento do estudo

Trata-se de um estudo epidemiológico do tipo transversal realizado na Região Metropolitana da Grande Vitória (RMGV) entre março e dezembro de 2023, em escolas de Ensino Médio públicas e privadas. 

### Local 

O estudo ocorreu na RMGV, constituída por: Cariacica, Fundão, Guarapari, Serra, Viana, Vila Velha e Vitória. A RMGV, segundo o Instituto Brasileiro de Geografia e Estatística (IBGE, dados de 2020), tem uma população de aproximadamente 1.880.828 pessoas e contém uma área de 281.760,93 km^2^. 

### Período

A coleta de dados ocorreu entre março e dezembro de 2023. 

### População

A população do estudo foi composta por adolescentes do Ensino Médio, de 14 a 19 anos, regularmente matriculados em 63 escolas públicas ou privadas da RMGV. Os responsáveis assinaram o Termo de Consentimento Livre e Esclarecido (TCLE) e o estudante o Termo de Assentimento Livre e Esclarecido (TALE). Como critério de exclusão, a falta de compreensão devido a déficit intelectual do participante, de forma que não haveria condições intelectuais para o aluno responder ao instrumento. 

### Definição da amostra

Para o cálculo da amostra, visando representatividade da rede pública e privada, a amostragem foi por conglomerado e estratificação. Assim, para cada estrato, foram realizadas amostras simples, sendo corrigidas para amostras complexas em um segundo momento usando SPSS 26, garantindo 95% de confiança e erro máximo de 5%. Ademais, para cada estrato de município, obteve-se como parâmetros da amostra 95% de confiança e erro máximo de 10%. 

Vale destacar que foi realizado o treinamento da equipe de pesquisa e o teste-piloto, que ocorreu no mês de fevereiro de 2023, no qual participaram 50 adolescentes que não compuseram a amostra final do estudo. O teste-piloto foi importante para a realização de pequenos ajustes, no que tange a questões sociodemográficas e comportamentais, de modo a promover maior clareza das questões, bem como foi fundamental para a estimativa do tempo de resposta ao questionário que foi em média de quarenta a cinquenta minutos. A coleta de dados ocorreu em *tablets* por meio do REDCap (*Research Electronic Data Capture*), com questões socioeconômicas (sexo, idade, raça, renda, situação familiar) e comportamentais (uso de cocaína, crack, *ecstasy* e metanfetamina). 

### Variáveis do estudo

A variável desfecho foi composta pelo uso de drogas estimulantes segundo o autorrelato do adolescente, as respostas foram dicotomizadas em sim e não, sendo considerado o uso de drogas estimulantes o adolescente que em pelo menos um dos itens apresentou relato positivo ao uso de alguma das seguintes drogas: cocaína, crack, *ecstasy* e/ou metanfetamina alguma vez na vida e uso atual. 

As variáveis independentes foram: sexo (feminino ou masculino), faixa etária (14 a 15 anos, 16 a 17 anos, 18 a 19 anos), identidade de gênero (cisgênero ou não cisgênero), orientação sexual (heterossexual ou LGBTQIA+), raça-cor (branca, preta e parda), em um relacionamento (sim ou não), possui algum trabalho, emprego ou negócio (sim ou não), classificação econômica (A; B; C/D/E), pais vivem juntos (sim ou não), tipo de escola (pública ou privada); religião (nenhuma; católica; evangélica; outras).

### Tratamento e análise dos dados

A análise estatística foi conduzida inicialmente por descrição dos dados através de apresentação de frequências absolutas, relativas e intervalos de confiança de 95% (IC95%), sendo considerado exclusão por caso completo, seguido de análise bivariada por teste Qui-quadrado de Pearson (χ²) e o teste Exato de Fisher, para verificar as possíveis relações entre as variáveis. Posteriormente, realizou-se análise multivariada bruta e ajustada de Razão de Prevalência (RP), por Regressão de Poisson com variância robusta, sendo considerada para entrada no ajuste as variáveis com significância estatística em 0,20 e mantidas aquelas com valor de p<0,05. Todas as análises foram realizadas através do programa estatístico Stata 17.0. 

### Aspectos éticos

O projeto foi submetido ao Comitê de Ética em Pesquisa da Universidade Federal do Espírito Santo, ocorrendo aprovação por meio do parecer n.° 5.900.370, de forma a atender à resolução n.°466/2012. Obteve-se a assinatura dos responsáveis pelo Termo de Consentimento Livre e Esclarecido (TCLE) e dos adolescentes no Termo de Assentimento Livre e Esclarecido (TALE). 

## Resultados

A prevalência de uso alguma vez na vida de alguma droga estimulante entre os escolares foi de 6% (IC95% 5,4-6,8), sendo que atualmente 1,6% usam alguma droga estimulante (IC95% 1,3-2,0). A prevalência de escolares que relataram já ter feito uso de cocaína alguma vez na vida foi de 1,2% (IC95% 1,0-1,6), e o uso atual dessa droga é de 0,2% (IC95% 0,1-0,4). Quanto ao uso de crack ou merla, a prevalência foi de 0,1% (N=6, IC95% 0,0-0,2), e o uso atual de 0,1% (IC95% 0,0-0,2). Aproximadamente 5% da amostra afirmou que já experimentou *ecstasy* ou bala ou MD na vida, e atualmente 1,4% fazem o uso dessa droga (IC95% 1,1-1,8). Referente ao uso de metanfetamina, 0,6% dos entrevistados declararam que já experimentaram na vida metanfetamina (IC95% 0,4-0,9) e 0,1% usam atualmente (IC95% 0,0-0,3) (Tabela 1).

**Tabela 1:** Uso de drogas estimulantes entre os escolares do Ensino Médio. Região Metropolitana da Grande Vitória, ES, Brasil, março a dezembro, 2023

	N*	%^†^	IC95%^‡^
Uso alguma vez na vida			
**Drogas estimulantes**			
Não	4334	94,0	93,2-94,6
Sim	278	6,0	5,4-6,8
**Cocaína**			
Não	4555	98,8	98,4-99,0
Sim	57	1,2	0,9-1,6
***Crack* ou merla**			
Não	4604	99,9	99,7-99,9
Sim	06	0,1	0,01-0,3
***Ecstasy***			
Não	4383	95,1	94,4-95,7
Sim	227	4,9	4,3-5,6
**Metanfetamina**			
Não	4581	99,4	99,1-99,6
Sim	29	0,6	0,4-0,9
**Uso atualmente**			
**Drogas estimulantes**			
Não	4538	98,4	97,9-98,7
Sim	74	1,6	1,3-2,0
**Cocaína**			
Não	4602	99,8	99,6-99,9
Sim	10	0,2	0,1-0,4
***Crack* ou merla**
Não	4607	99,9	99,8-100,0
Sim	3	0,1	0,0-0,2
***Ecstasy***
Não	4544	98,6	98,2-98,9
Sim	66	1,4	1,1-1,8
**Metanfetamina**
Não	4603	99,9	99,7-99,9
Sim	7	0,1	0,0-0,3

*N = Frequência absoluta; ^†^% = Prevalência; ^‡^IC95% = Intervalo de confiança de 95%

De acordo com a Tabela 2, a idade de uso de drogas estimulantes pela primeira vez foi de 15 anos ou mais para todas as substâncias analisadas, sendo que a proporção de início de uso a partir dessa idade foi de 50% para a cocaína (IC95% 36,7-63,3), 50% para o *crack* (IC95% 10,9-89,1), 77,9% para o *ecstasy* (IC95% 72,0-82,8) e 64% para o uso de metanfetamina (IC95% 42,9-80,8). Ainda, de acordo com os participantes, 32,1% conseguiram a cocaína com um amigo (IC95% 21,1-45,7), e, do mesmo modo, o *ecstasy* foi adquirido por meio de amigos para cerca de 39% dos adolescentes (IC95% 33,0-45,7). Por outro lado, crack/merla apresentaram maior frequência de aquisição em lojas, bares, botequim, com um vendedor e/ou na balada (50%; IC95% 10,9-89,1), e, por fim, cerca de 28% dos adolescentes compraram e/ou conseguiram a metanfetamina na balada (13,8%, IC95% 5,0-32,5) ou com amigos (13,8%, IC95% 5,0-32,5). Vale destacar para todas as drogas a elevada prevalência de adolescentes que referem não se recordarem de como a substância foi adquirida.

**Tabela 2:** Uso de drogas estimulantes entre os escolares do Ensino Médio. Região Metropolitana da Grande Vitória, ES, Brasil, março a dezembro, 2023

	Cocaína			*Crack*/merla			*Ecstasy*/bala/MD			Metanfetamina		
	N*	%^†^	IC95%^‡^	N*	%^†^	IC95%^‡^	N*	%^†^	IC95%^‡^	N*	%^†^	IC95%^‡^
Que idade você tinha quando experimentou pela primeira vez?												
Menos de 10 anos	10	18,5	10,1-31,5	2	33,3	5,1-82,2	7	3,1	1,5-6,4	3	12,0	3,7-32,7
11 a 14 anos	17	30,5	20,3-45,3	1	16,7	1,2-77,0	43	19,0	14,4-24,7	6	24,0	10,7-45,4
15 ou mais	27	50,0	36,7-63,3	3	50,0	10,9-89,1	176	77,9	72,0-82,8	16	64,0	42,9-80,8
**Como você conseguiu?**												
Comprei numa loja, bar, botequim, com um vendedor e/ou na balada	9	16,1	8,5-28,4	3	50,0	10,9-89,1	54	23,8	18,7-29,8	4	13,8	5,0-32,5
Dei dinheiro para alguém comprar para mim	4	7,1	2,6-17,9	1	16,7*	1,2-77,0	9	4,0	2,1-7,5	-	-	-
Peguei escondido em casa	3	5,4	1,7-15,7	-	-	-	-	-	-	2	6,9	1,2-25,0
Uma pessoa mais velha me deu	1	1,8	2,4-12,1	-	-	-	21	9,3	6,1-13,8	-	-	-
Alguém da minha família me deu	1	1,8	2,4-12,1	1	16,7*	1,2-77,0	4	1,8	0,7-4,2	3	10,3	3,2-28,7
Consegui com um amigo	18	32,1	21,1-45,7	-	-	-	89	39,2	33,0-45,7	4	13,8	5,0-32,5
Consegui na escola	-	-	-	-	-	-	-	-	-	1	3,5	0,4-22,3
Não lembro e outro modo	20	35,7	24,1-49,3	1	16,7*	14,3	50	22,0	17,1-27,9	15	51,7	33,4-69,6

Nota: sem possibilidade de arredondar, percentual acima; *N = Frequência absoluta; ^†^% = Prevalência; ^‡^IC95% = Intervalo de confiança de 95%

A Tabela 3 apresenta a distribuição do uso de drogas estimulantes entre os escolares do Ensino Médio, segundo as características em estudo. Nota-se uma relação entre a experimentação e o uso de estimulantes e as variáveis: identidade de gênero, orientação sexual, raça/cor da pele, estado civil, tipo de escola, se possui trabalho atualmente e religião (p<0,05). Já as variáveis idade e situação conjugal dos pais somente apresentaram relação significativa com a experimentação dos estimulantes (p<0,001), e o uso atual da droga com o sexo e classe econômica (p<0,05).

**Tabela 3:** Distribuição do uso de drogas estimulantes entre os escolares do Ensino Médio da Região Metropolitana da Grande Vitória. ES, Brasil, março a dezembro, 2023

Variáveis	Experimentação de estimulantes				Uso atual de estimulantes			
	N*	%^†^	IC95%^‡^	p-valor	N*	%^†^	IC95%^‡^	p-valor
**Sexo^§^**				0,137				0,009
Feminino	139	5,5	4,72-6,52		29	1,2	0,81-1,66	
Masculino	139	6,6	5,61-7,74		45	2,1	1,60-2,84	
**Idade (anos)**				<0,001				0,248
14 a 15	37	3,8	2,3-5,2		12	1,2	0,70-2,27	
16 a 17	179	5,9	5,1-6,8		48	1,6	1,29-2,09	
18 a 19	62	10,3	8,0-13,0		14	2,3	1,38-3,88	
**Identidade de gênero**				0,032				0,010
Cisgênero	254	5,8	5,18-6,58		64	1,5	1,16-1,89	
Não cisgênero	26	8,9	6,15-12,8		10	3,4	1,86-6,27	
**Orientação sexual**				<0,001				0,049
Heterossexual	193	5,3	4,59-6,04		52	1,4	1,10-1,86	
LGBTQIA+	85	9,0	7,31-11,00		22	2,3	1,53-3,50	
**Raça/Cor da pele**				0,001				0,003*
Branca(o)	83	4,7	3,8-5,8		16	0,9	0,60-1,48	
Preta(o)	66	8,7	6,9-10,9		21	2,8	1,81-4,20	
Parda(o)	113	5,8	4,8-6,3		30	1,5	1,08-2,19	
**Estado civil**				0,001				0,006
Com companheiro	98	8,0	6,59-9,63		44	1,3	0,97-1,74	
Sem companheiro	180	5,3	4,61-6,12		30	2,4	1,71-3,47	
**Classificação socioeconômica**				0,957				0,017*
A	497	6,2	4,89-8,40		10	1,3	0,71-2,42	
B	129	5,9	5,05-7,05		47	2,2	1,62-2,86	
C//D/E	102	6,1	5,12-7,44		17	1,0	0,63-1,63	
**Tipo de escola**				<0,001				0,019
Pública	217	7,0	6,17-8,00		59	1,9	1,48-2,46	
Privada	61	4,0	3,13-5,12		15	1,0	0,59-1,63	
**Atualmente, possui algum trabalho, emprego ou negócio**				<0,001				<0,001
Não	176	5,04	4,36-5,81		40	1,1	0,84-1,56	
Sim	102	9,12	7,58-11,00		31	3,0	2,18-4,23	
**Religião**				<0,001				<0,001
Nenhuma	91	8,4	6,85-10,15		25	2,3	1,56-3,38	
Católica	55	4,7	3,60-6,33		11	0,9	0,52-1,68	
Evangélica	89	4,4	3,62-4,43		22	1,1	0,72-1,66	
Outras	43	12,7	9,57-16,72		16	4,7	2,92-7,59	
**Situação conjugal dos pais - vivem juntos**				<0,001				0,056
Sim	99	4,10	3,37-4,96		29	1,2	0,83-1,72	
Não	174	8,04	7,00-9,26		41	1,9	1,40-2,56	

*N = Frequência absoluta; ^†^% = Prevalência; ^‡^IC95% = Intervalo de confiança de 95%; ^§^Exato de Fisher

Após ajuste para os fatores de confusão, observa-se que aqueles com 18 a 19 anos de idade tiveram uma prevalência 2,24 maior (IC95% 1,47-3,40) de experimentação de drogas estimulantes em comparação aos adolescentes de 14 a 15 anos. Escolares que se autodeclararam LGBTQIA+ tiveram 35% mais prevalência de experimentação (IC95% 1,04-1,77) em comparação aos heterossexuais, assim como os de raça-cor preta apresentaram uma prevalência de 1,55 vezes maior de experimentação de drogas estimulantes (IC95% 1,13-2,14) em comparação aos adolescentes da raça-cor branca. A experimentação alguma vez na vida de estimulantes foi 68% mais prevalente entre os escolares com trabalho (IC95% 1,32-2,13). Pertencer à religião católica reduziu a prevalência de experimentação de drogas estimulantes em 30% (RP: 0,70; IC95% 0,50-0,99), como também na religião evangélica, apresentando redução em 41% (RP: 0,59; IC95% 0,44-0,81) em comparação aos adolescentes que declararam não ter nenhuma religião. Por fim, adolescentes cujos pais vivem separados tiveram uma frequência de 62% maior de experimentação de drogas estimulantes (IC95% 1,26-2,10) em comparação àqueles cujos pais vivem juntos (Tabela 4).

**Tabela 4:** Razão de prevalência bruta e ajustada da experimentação de drogas estimulantes entre os escolares do Ensino Médio. Região Metropolitana da Grande Vitória, ES, Brasil, março a dezembro, 2023

Variáveis	Experimentação de drogas estimulantes					
	RP bruta*	IC95%^†^	p-valor	RP ajustada^‡^	IC95%^†^	p-valor
**Sexo**			0,138			0,155
Feminino	1,0			1,0		
Masculino	1,19	0,95-1,49		1,20	0,93-1,53	
**Idade (anos)**			<0,001			<0,001
14 a 15	1,0			1,0		
16 a 17	1,55	1,09-2,19		1,48	1,03-2,13	
18 a 19	2,69	1,81-3,99		2,24	1,47-3,40	
**Identidade de gênero**			0,031			0,862
Cisgênero	1,0			1,0		
Não cisgênero	1,53	1,04-2,25		0,96	0,61-1,51	
**Orientação sexual**			<0,001			0,027
Heterossexual	1,0			1,0		
LGBTQIA+	1,70	1,33-2,17		1,35	1,04-1,77	
**Raça-cor da pele**			<0,001			0,025
Branca(o)	1,0			1,0		
Preta(o)	1,86	1,36-2,54		1,55	1,13-2,14	
Parda(o)	1,24	0,94-1,63		1,19	0,90-1,58	
**Estado civil**			0,001			0,063
Com companheiro	1,50	1,18-1,90		1,27	0,99-1,63	
Sem companheiro	1,0			1,0		
**Classificação socioeconômica**			0,957			-
A	1,01			-	-	
B	0,98	0,75-1,25		-	-	
C//D/E	1,0	0,72-1,41		-	-	
**Tipo de escola**			<0,001			0,177
Pública	1,75	1,33-3,31		1,24	0,91-1,68	
Privada	1,0			1,0		
**Atualmente, possui algum trabalho, emprego ou negócio**			<0,001			<0,001
Não	1,0			1,0		
Sim	1,81	1,43-2,29		1,68	1,32-2,13	
**Religião**			<0,001			<0,001
Nenhuma	1,0			1,0		
Católica	0,56	0,40-0,77		0,70	0,50-0,99	
Evangélica	0,53	0,40-0,70		0,59	0,44-0,81	
Outras	1,52	1,08-2,14		1,36	0,94-1,95	
**Situação conjugal dos pais - vivem juntos**			<0,001			<0,001
Sim	1,0			1,0		
Não	1,96	1,53-2,51		1,62	1,26-2,10	

*RP bruta = Razão de prevalência bruta; ^†^IC95% = Intervalo de confiança de 95%; ^‡^RP ajustada = Razão de prevalência ajustada

O uso atual de drogas estimulantes após ajuste dos dados foi mais prevalente para adolescentes da raça-cor preta (RP 2,94; IC95% 1,53-5,65) em comparação aos adolescentes da raça-cor branca. Comparado aos adolescentes que pertencem a classificações econômicas mais baixas (C/D/E), adolescentes pertencentes à classe B tiveram uma prevalência 2,27 vezes maior no uso atual de drogas estimulantes (IC95% 1,31-3,95). Possuir um trabalho levou a uma prevalência 2,69 vezes maior no uso atual (IC95% 1,69-4,30) em comparação aos adolescentes sem trabalho, e a prevalência de uso atual de drogas estimulantes foi 58% menor naqueles pertencentes à religião católica (IC95% 0,20-0,87) e 57% menor nos escolares evangélicos (IC95% 0,24-0,78), em comparação aos adolescentes que relataram não ter religião (Tabela 5).

**Tabela 5:** Análise bruta e ajustada do uso atual de drogas estimulantes entre os escolares do Ensino Médio. Região Metropolitana da Grande Vitória, ES, Brasil, março a dezembro, 2023

Variáveis	Uso atual de drogas estimulantes					
	RP bruto*	IC95%^†^	p-valor	RP ajustada^‡^	IC95%^†^	p-valor
**Sexo**			0,010			0,103
Feminino	1,0			1,0		
Masculino	1,84	1,16-2,93		1,49	0,92-2,41	
**Idade (anos)**			0,254			-
14 a 15	1,0			-	-	
16 a 17	1,28	0,68-2,40		-	-	
18 a 19	1,87	0,87-4,02		-	-	
**Identidade de gênero**			0,012			0,445
Cisgênero	1,0			1,0		
Não cisgênero	2,32	1,20-4,47		1,39	0,59-3,28	
**Orientação sexual**			0,050			0,223
Heterossexual	1,0			1,0		
Não Heterossexual	1,63	1,00-2,68		1,45	0,80-2,64	
**Raça/Cor da pele**			0,003			0,005
Branca(o)	1,0			1,0		
Preta(o)	3,06	1,61-5,84		2,94		
Parda(o)	1,71	0,93-3,12		1,74	1,53-5,65	
**Estado civil**			0,007		0,97-3,12	0,098
Sem companheiro	1,0			1,0		
Com companheiro	1,89	1,19-2,97		1,52	0,93-2,49	
**Classificação socioeconômica**			0,019			0,009
A	2,12	0,59-2,79		1,26	0,52-3,03	
B	1,29	1,22-3,67		2,27	1,31-3,95	
C//D/E	1,0			1,0		
**Tipo de escola**			0,021			0,672
Pública	1,94	1,10-3,40		1,14	0,61-2,13	
Privada	1,0			1,0		
**Atualmente, possui algum trabalho, emprego ou negócio**			<0,001			<0,001
Não	1,0			1,0		
Sim	2,66	1,69-4,18		2,69	1,69-4,30	
**Religião**			<0,001			<0,001
Nenhuma	1,0			1,0		
Católica	0,41	0,20-0,82		0,42	0,20-0,87	
Evangélica	0,48	0,27-0,84		0.43	0,24-0,78	
Espírita	2,06	1,11-3,82		1,49	0,75-2,94	
**Situação conjugal dos pais - vivem juntos**			0,060			0,395
Sim	1,0			1,0		
Não	1,58	0,98-2,54		1,24	0,76-2,03	

*RP bruta = Razão de prevalência bruta; ^†^IC95% = Intervalo de confiança de 95%; ^‡^RP ajustada = Razão de prevalência ajustada

## Discussão

A prevalência do uso de drogas estimulantes alguma vez na vida entre os escolares deste estudo foi de 6% (IC95%: 5,4-6,8), enquanto o uso atual foi de 1,6% (IC95%: 1,3-2,0). Esses achados estão em consonância com os dados divulgados pelo estudo norte-americano *Monitoring the Future* (MTF, 2022), que investigou adolescentes do Ensino Médio com até 18 anos, identificando prevalência semelhante de uso alguma vez na vida (6,1%) e de uso atual (1,5%) de drogas estimulantes. No mesmo sentido, também foi observado em estudo realizado no Chile com adolescentes de 13-17 anos, uma prevalência de 5,9% de uso de estimulantes alguma vez na vida e 1,7% de uso atual^18^.

No que tange ao uso de cocaína, observou-se que a prevalência de experimentação na vida entre os escolares foi de 1,2% (IC95%: 1,0-1,6). Esse resultado está em consonância com estudo realizado na Nigéria, no qual 1,4% dos adolescentes relataram uso dessa substância ao longo da vida^19^. De forma semelhante, pesquisa conduzida em Macau, China, identificou prevalência de 1,2% de uso de cocaína alguma vez na vida entre adolescentes^20^. Em contrapartida, estudo realizado no Brasil, no município de Cuiabá, encontrou prevalência superior, de 3,1%, de uso de cocaína ao longo da vida^21^. No que tange ao uso atual de cocaína, observou-se uma prevalência de 0,2% (IC95%: 0,1-0,4). Esse resultado é semelhante ao descrito em estudos com população adolescente, nos quais 0,4% dos adolescentes relataram uso atual de cocaína^19^. 

Quanto ao consumo de *crack* ou merla, a prevalência encontrada foi de 0,1% (IC95% 0,0-0,2). Esse resultado é inferior ao identificado pelo Projeto de Pesquisa Escolar Europeia sobre Álcool e Outras Drogas, que analisou dados de 99.647 adolescentes em 35 países e encontrou prevalência média de 1,1% de uso de *crack* alguma vez na vida. Nesse estudo, a maior prevalência foi observada no Chipre (3,1%), enquanto as menores ocorreram na Macedônia do Norte (0,2%) e na Eslováquia (0,5%)^9^. De forma semelhante, estudo transversal realizado no Brasil, no município de Cuiabá (MT), identificou prevalência de 1,4% de uso de *crack* alguma vez na vida entre adolescentes^21^.

No que se refere ao uso de *ecstasy*, bala ou MD, a prevalência alguma vez na vida foi de 5%, semelhante ao identificado no estudo *European School Survey Project on Alcohol and Other Drugs* (ESPAD)^9^, que revelou entre adolescentes do Ensino Médio na Holanda uma prevalência de 5,1%. Da mesma maneira, a prevalência de uso atual encontrada (1,4%; IC95% 1,1-1,8) vai ao encontro da registrada no ESPAD^9^, mas com foco nos adolescentes da República Tcheca, onde 1,7% relataram consumo dessas drogas.

A prevalência do uso de metanfetamina alguma vez na vida entre os escolares foi de 0,6% (IC95%: 0,4-0,9), valor superior, embora não distante, ao observado no estudo do Centro Brasileiro de Informações sobre Drogas Psicotrópicas (CEBRID, dados de 2020), que identificou prevalência inferior a 0,1% para o uso dessa substância. Ademais, o uso atual de metanfetamina neste estudo foi de 0,1% (IC95%: 0,0-0,3), indicando baixo consumo entre escolares, resultado semelhante ao descrito pelo ESPAD (2019), que apontou prevalência de 0,3% entre adolescentes^9^. 

A análise da idade de início do uso de drogas estimulantes revela uma tendência preocupante de que, para todas as substâncias analisadas, a primeira experiência ocorre a partir dos 15 anos. Cerca de 27,9% dos indivíduos iniciam o uso de drogas estimulantes antes dos 18 anos, com uma tendência mais pronunciada entre os homens, o que pode refletir diferenças sociais, culturais e biológicas na propensão ao uso^15^. O início precoce do uso dessas substâncias está intimamente associado ao desenvolvimento de problemas mais severos relacionados ao abuso e dependência de drogas na vida adulta^16^. 

Os dados da pesquisa indicam que a aquisição dos estimulantes se dá por meio de amigos ou por compras em lojas, bares, botequins, com um vendedor e/ou na balada. Estudos relataram maior frequência de jovens adquirindo drogas estimulantes (*ecstasy*, anfetamina, cocaína) em bares, casas noturnas, festas e etc.^22^ ^-^ ^25^. É provável que isso ocorra, pois em festas, tais como, *raves,* são mais associadas a maiores chances do uso dessas drogas, uma vez que drogas usadas em tais festas para melhorar a música, as luzes, a socialização e a dança a noite toda^22^. 

Outro achado foi a maior prevalência de experimentação dos estimulantes entre adolescentes mais velhos com 18 a 19 anos de idade (IC95% 1,47-3,40). Assim como os achados no presente estudo, identificou-se que o uso desse tipo de drogas é feito pelos jovens mais velhos^14^. Além disso, há relatos na literatura de uma idade média de 18-19 anos para *cannabis* e 21-24 anos para cocaína^26^. Ademais, entre os anos de 2000 e 2001, já havia evidências de grande crescimento de uso de estimulantes do tipo anfetamina entre adolescentes na Ásia e Europa^27^. 

Ainda, observa-se a experimentação dos estimulantes maior entre os jovens que se autodeclararam LGBTQIA+, com 35% mais prevalência (IC95% 1,04-1,77). Esse dado é similar ao de outras pesquisas, que sugerem que esse grupo populacional corre mais risco de uso dessas substâncias relacionado a estressores minoritários, como o estigma sofrido^28^ ^-^ ^29^.

Ainda, em nosso estudo, verifica-se a maior frequência de experimentação e uso atual dos estimulantes entre pessoas de raça/cor preta, dados ainda pouco explorados na literatura. Um estudo realizado nos Estados Unidos mostra que jovens de cor branca estão mais propensos a receber tratamento, e têm mais acesso a serviços de saúde privados^30^. 

O presente estudo encontrou maior prevalência da experimentação e uso atual de estimulantes entre os escolares que trabalhavam, dados em consonância com pesquisa que utilizou dados de mais de 109 mil alunos brasileiros e encontrou maior frequência de uso de drogas entre os adolescentes que trabalham, e sugere que o trabalho remunerado pode possibilitar maior independência financeira e influenciar a autonomia do adolescente na tomada de decisão para o consumo de drogas^31^.

A associação entre o uso de estimulantes e marcadores sociais como raça/cor e o pertencimento a grupos socialmente estigmatizados, como pessoas LGBTQIA+, evidencia o papel das desigualdades estruturais na produção de vulnerabilidades em saúde. O racismo estrutural e o preconceito atuam como determinantes sociais que ampliam a exposição a estressores crônicos, fragilizam redes de apoio e restringem o acesso oportuno a serviços de saúde e de proteção social, especialmente durante a adolescência^32^. Nesse contexto, a teoria do estresse de minorias trata-se de uma abordagem interseccional, segundo a qual a sobreposição de múltiplos marcadores de minoria intensifica a exposição a fatores de risco e potencializa impactos negativos sobre a saúde mental. Ela sustenta que experiências persistentes de estigmatização, discriminação e violência aumentam o risco de sofrimento psíquico e do uso de substâncias como estratégia de enfrentamento^33^.

Pertencer à religião católica ou evangélica diminuiu a prevalência de experimentação e uso atual de drogas estimulantes em comparação aos adolescentes que declararam não ter nenhuma religião. Os achados corroboram um estudo transversal realizado no interior de São Paulo no qual os escolares que praticavam uma religião apresentaram menor chance de usar drogas^34^. A religiosidade suscita uma mistura de medo e tabu, e, na forma de crenças e práticas, pode ser vista como um forte fator de resiliência e resistência contra o uso de substâncias pelos adolescentes^35^. 

Outro achado foi o uso atual mais frequente nos adolescentes que pertencem a classificações econômicas mais altas. Uma pesquisa transversal na Nigéria, com mais de mil adolescentes em escolas públicas e privadas, encontrou que estudantes que frequentam escolas em áreas povoadas por pessoas com renda mais alta tiveram maior probabilidade de abusar de drogas, entre elas a cocaína. É possível que haja uma relação com o maior poder de compra disponível para os estudantes nessas áreas^19^. 

Verificou-se ainda que adolescentes em que os pais vivem separados tiveram uma frequência de 62% maior de experimentação de drogas estimulantes (IC95% 1,26-2,10) em comparação àqueles cujos pais vivem juntos. Este resultado está em consonância com um estudo realizado nos Estados Unidos com dados de mais de 41 mil adolescentes, no qual aqueles pertencentes a famílias monoparentais e não parentais tiveram uma probabilidade maior de usar cocaína/heroína/metanfetamina (OR = 1,63-1,78, IC 95% = 1,21-2,71) do que os jovens em famílias biparentais^36^. Uma das possíveis justificativas seria que as famílias com dois pais são mais propensas a monitorar os jovens de perto, dessa forma estes teriam menos chance de se expor a comportamentos de risco^36^. Além disso, para além da relação dos pais, o tempo dedicado aos filhos pode influenciar no consumo de substâncias psicoativas^37^. 

Considerando os resultados apresentados e os impactos do uso de substâncias psicoativas para a saúde, os profissionais de enfermagem possuem um importante papel neste cenário. O cuidado de enfermagem a adolescentes que fazem uso de drogas ou possuem forte risco de fazer o uso, deve ser pautado em uma atitude acolhedora, sem preconceitos e capaz de propiciar um espaço de diálogo. Devem ser utilizadas estratégias de promoção e prevenção à saúde, guiadas nas melhores evidências científicas, com foco na autonomia do sujeito e redução de danos^38^. As ações podem ser realizadas na própria consulta de Enfermagem individual ou em atividades coletivas. Dessa forma, é possível traçar estratégias para torná-los mais reflexivos, competentes e confiantes. Quando as potencialidades são estimuladas, há um fortalecimento da autoestima, de modo a possibilitar escolhas e decisões conscientes^39^. 

O delineamento transversal apresenta limitações no que se refere à determinação da causalidade, uma vez que o desfecho é avaliado ao mesmo tempo que a exposição, e isso impede a identificação da temporalidade dos eventos, sendo necessário cautela ao se avaliar as relações entre as variáveis exploradas. Ainda, o viés de informação também pode ocorrer, comprometendo a precisão das respostas obtidas, entretanto, vale ponderar que os estudantes puderam responder ao questionário de forma individualizada, respeitando a privacidade deles, reduzindo então o viés supracitado. 

## Conclusão

Podemos concluir que 6% dos adolescentes do Ensino Médio já fizeram uso, alguma vez na vida, de drogas estimulantes e que 1,6% usam atualmente. A idade de uso pela primeira vez, para a maioria, foi aos 15 anos ou mais, e, a aquisição dessa substância acontece, principalmente, em lojas, bares, botequins, com um vendedor e/ou na balada. Maiores prevalências de experimentação foram observadas entre adolescentes mais velhos (18 a 19 anos) que se autodeclararam LGBTQIA+, de raça-cor preta, escolares com trabalho e cujos pais vivem separados. O uso atual mais frequente entre os pertencentes à classe B, de raça-cor preta, e que trabalham. Aqueles que possuem religião (evangélica ou católica) apresentaram menores prevalências de experimentação e uso atual. 

Os achados da presente pesquisa reforçam a necessidade de estratégias de enfrentamento contra o uso de drogas estimulantes entre adolescentes no contexto comunitário, familiar e escolar. Esforços para a articulação intersetorial entre saúde, educação, assistência social e segurança pública, assim como inserir a família nesse processo, devem ser empregados para prevenção e mitigação do uso. No contexto acadêmico, é fundamental a realização de estudos na abordagem qualitativa que explore principalmente os motivos relacionados ao consumo maior entre esses adolescentes, além do desenvolvimento de pesquisas de intervenções baseadas, com o objetivo de fortalecer as ações de prevenção. Por fim, é necessário potencializar os fatores que reduzem a exposição ao risco do uso dessas substâncias a fim de promover uma juventude saudável.

## Data Availability

Os conjuntos de dados relacionados a este artigo estarão disponíveis mediante solicitação ao autor correspondente.

# Translation: Uso de drogas estimulantes entre adolescentes de educación secundaria en una Región Metropolitana: un estudio con base escolar^*^

**DOI:** 10.1590/1518-8345.8157.4963

**Authors:** Franciéle Marabotti Costa Leite, Tiffani Matos Oliveira, Letícia Peisino Buleriano, Micael Franco Alves, Mayara Alves Luis, Carlos Augusto Lopes, Fernanda Garcia Gabira Miguez

**Affiliations:** 1 Universidade Federal do Espirito Santo, Vitória, ES, Brasil.; 2 Prefeitura de Vitória, Secretaria Municipal de Saúde de Vitória, Vitória, ES, Brasil.

**Keywords:** Adolescentes, Estimulantes del Sistema Nervioso Central, Prevalencia, Factores de Riesgo, Estudios Transversales, Minorías Sexuales y de Género.

## Abstract

**Objetivo:**

identificar la prevalencia de los estimulantes entre adolescentes en educación secundaria y los factores asociados.

**Método:**

estudio transversal con 4.614 adolescentes de entre 14 y 19 años. Se realizaron análisis descriptivos, bivariantes (prueba del qui-cuadrado y test exacto de Fisher) y multivariantes (regresión de Poisson con varianza robusta), adoptando un nivel de significación de p<0,05.

**Resultados:**

la prevalencia de consumo a lo largo de la vida fue del 6% (intervalo de confianza del 95%: 5,4-6,8) y el uso actual fue del 1,6% (intervalo de confianza del 95%: 1,3-2,0). Mayor prevalencia de experimentación con drogas estimulantes entre adolescentes de 18 a 19 años (RP: 2,24), LGBTQIA+ (RP: 1,35), personas de raza/color de piel negra (RP: 1,55), con trabajo (RP: 1,68) y cuyos padres viven separados (RP: 1,62). Los adolescentes que pertenecían a la religión católica o evangélica tenían menores frecuencias de experimentación y uso actual (p<0,05). El uso actual fue más frecuente entre personas de raza negra (RP 2,94), clase B (RP: 2,27) y quienes tienen empleo (RP: 2,69).

**Conclusión:**

se observa que la experimentación y el uso de drogas estimulantes están presentes entre los adolescentes y que factores sociodemográficos y conductuales están asociados a esta práctica. Son necesarias estrategias integradas entre los sectores de salud, educación, asistencia social y seguridad pública para la prevención y mitigación del problema en entornos escolares, familiares y comunitarios.

## Destacados

**(1)** El estudio investigó el consumo de drogas estimulantes entre adolescentes de educación secundaria.

**(2)** La prevalencia de consumo a lo largo de la vida fue del 6% y el consumo actual del 1,6%.

**(3)** La edad avanzada, el trabajo, la raza-color negro y ser LGBTQIA+ aumentaron la prevalencia.

**(4)** La religión católica o evangélica mostró un efecto protector contra el uso de estimulantes.

**(5)** Los hallazgos respaldan acciones intersectoriales de prevención en el contexto escolar y comunitario.

## Introducción

La Organización Mundial de la Salud (OMS) define las drogas como cualquier sustancia no producida por el cuerpo que, al introducirse en el organismo, es capaz de actuar sobre uno o más sistemas, provocando cambios en su funcionamiento^1^. En este contexto, las drogas psicoactivas se caracterizan por su capacidad para afectar los procesos mentales y cognitivos de las personas, influyendo en las percepciones, emociones, comportamiento y conciencia^2^.

Históricamente, los seres humanos han seguido interesados en sustancias psicoactivas, y el primer contacto ha ocurrido a una edad cada vez más temprana, como en la adolescencia, que es un periodo de gran vulnerabilidad, ya que es un periodo de búsqueda de identidad, mayor necesidad de aceptación social y tendencia a poner a prueba límites^3^ ^-^ ^5^.

Además, la inmadurez cerebral, especialmente en regiones relacionadas con el autocontrol y la toma de decisiones, también contribuye a la impulsividad, lo que puede aumentar la vulnerabilidad a conductas de riesgo. Este escenario representa una preocupación creciente en el contexto de la salud pública, ya que afecta directamente a la salud física, emocional y social de los adolescentes^6^.

En cuanto a las estimaciones, la evidencia internacional apunta a una prevalencia relevante del consumo de drogas estimulantes entre los adolescentes. Un estudio realizado con 33.184 adolescentes en cinco países del sudeste asiático identificó que la prevalencia del consumo de anfetaminas a lo largo de la vida oscilaba entre el 1,2% en Laos y el 4,8% en Filipinas y el 5,1% en Timor-Leste^7^. En Estados Unidos, los datos de la Encuesta de Conducta de Riesgo Juvenil (YRBS) indicaron que el 5,7% de los adolescentes entrevistados reportaron consumo de cocaína en 2015^8^. En Europa, se observa que el 2% de los adolescentes había probado la cocaína al menos una vez^9^. En Australia, un estudio indica que el 7,3% de las personas entre 14 y 24 años reportaron consumo de cocaína, mientras que el 7,6% indicó haber consumido éxtasis en los 12 meses previos a la encuesta^10^. 

En Brasil, un estudio realizado con 271 estudiantes en el interior de Pernambuco mostró que el 19,6% de los estudiantes reportó haber consumido alguna sustancia psicoactiva en sus vidas y/o en los treinta días previos a la encuesta^5^. La II Encuesta Nacional sobre el consumo de drogas por parte de la población brasileña (2019) indicó que el 2,5% de los adolescentes entre 13 y 15 años reportó consumo de cocaína^11^. Por otro lado, la encuesta PeNSE (2019), realizada por el Ministerio de Salud, indicó que el 1,2% de los adolescentes entre 13 y 17 años ya había probado esta droga^12^, mientras que el 1,6% reportó consumo de éxtasis, con una prevalencia ligeramente menor de consumo de crack y metanfetamina^12^. Estos hallazgos corroboran las estimaciones de la Oficina de las Naciones Unidas contra la Droga y el Delito (UNODC), que apuntan a un aumento progresivo en el uso de estas drogas, especialmente en los grandes centros urbanos.

Además, el consumo de drogas psicoactivos genera una mayor sensación de bienestar, euforia, excitación y alerta, así como un aumento de la actividad motora. Sin embargo, también reducen el apetito y pueden provocar insomnio, cambios cardiovasculares, mayor exposición a accidentes y violencia^13^ ^-^ ^15^. Además, el consumo puede estar asociado con un bajo rendimiento escolar, absentismo y abandono escolar, y una mayor implicación en conductas de infracción y situaciones de vulnerabilidad social, con posibles repercusiones a lo largo de la vida adulta. En dosis altas, pueden provocar inquietud, agitación, percepciones alteradas de la realidad y alucinaciones. Además, los efectos del consumo crónico y la abstinencia de estas sustancias pueden incluir paranoia, psicosis, depresión y/o ideación suicida^14^ ^-^ ^17^. En las relaciones familiares, tiene repercusiones negativas, fomentando conflictos familiares y debilitando los lazos de apoyo^14^ ^-^ ^16^.

En vista de lo anterior, el uso de drogas estimulantes entre adolescentes es un importante problema de salud pública y, en este sentido, los profesionales sanitarios, especialmente las enfermeras que trabajan en diferentes niveles de atención a la salud, desempeñan un papel estratégico en la prevención, promoción y acciones de educación en salud en entornos escolares, familiares y comunitarios. Sin embargo, a pesar de la relevancia del tema, existen muchas lagunas en la literatura científica, especialmente en lo que respecta a prevalencias regionales y grupos de mayor vulnerabilidad, un hecho que justifica el presente estudio, que pretende identificar la prevalencia de drogas estimulantes entre adolescentes en educación secundaria y los factores asociados.

## Método

### Diseño del estudio

Se trata de un estudio epidemiológico transversal realizado en la Región Metropolitana de la Gran Vitória (RMGV) entre marzo y diciembre de 2023, tanto en institutos públicos como privados.

### Lugar

El estudio se llevó a cabo en la RMGV, compuesta por: Cariacica, Fundão, Guarapari, Serra, Viana, Vila Velha y Vitória. La RMGV, según el Instituto Brasileño de Geografía y Estadística (IBGE, datos de 2020), tiene una población de aproximadamente 1.880.828 personas y contiene una superficie de 281.760,93 km^2^. 

### Periodo

La recogida de datos tuvo lugar entre marzo y diciembre de 2023.

### Población

La población estudiada estaba compuesta por adolescentes de educación secundaria de entre 14 y 19 años, matriculados regularmente en 63 escuelas públicas o privadas del RMGV. Los padres o tutores firmaron el Formulario de Consentimiento Informado (FCI) y el estudiante el Formulario de Asentimiento Libre e Informado (TALE). Como criterio de exclusión, la falta de comprensión debida al déficit intelectual del participante, de modo que no habría condiciones intelectuales para que el estudiante respondiera al instrumento.

### Definición de la muestra

Para calcular la muestra, buscando la representatividad de las redes públicas y privadas, el muestreo se realizó por conglomerado y estratificación. Así, para cada estrato se realizaron muestras simples, corrigiéndose para muestras complejas en un segundo momento usando SPSS 26, asegurando un 95% de confianza y un error máximo del 5%. Además, para cada estrato del municipio, los parámetros de la muestra tenían un 95% de confianza y un error máximo del 10%.

Cabe destacar que la formación del equipo de investigación y la prueba piloto se llevaron a cabo, que tuvo lugar en febrero de 2023, en la que participaron 50 adolescentes que no formaban la muestra final del estudio. La prueba piloto fue importante para realizar pequeños ajustes en cuestiones sociodemográficas y de comportamiento, con el fin de promover una mayor claridad en las preguntas, además de ser fundamental para estimar el tiempo de respuesta al cuestionario, que era de media entre cuarenta y cincuenta minutos. La recogida de datos se realizó en tabletas utilizando REDCap (Captura Electrónica de Datos de Investigación), con cuestiones socioeconómicas (género, edad, raza, ingresos, situación familiar) y de comportamiento (consumo de cocaína, crack, éxtasis y metanfetamina).

### Variables del estudio

La variable de resultado consistió en el uso de drogas estimulantes según el autoinforme de los adolescentes, las respuestas se dichotomizaron en sí y no, y el uso de drogas estimulantes se consideró el adolescente que, en al menos uno de los ítems, presentó un informe positivo de consumo de alguna de las siguientes drogas: cocaína, crack, éxtasis y/o metanfetamina al menos una vez en la vida y uso actual. 

Las variables independientes eran: sexo (mujer o hombre), grupo de edad (14 a 15 años, 16 a 17 años, 18 a 19 años), identidad de género (cisgénero o no cisgénero), orientación sexual (heterosexual o LGBTQIA+), raza-color (blanco, negro y moreno), en una relación (sí o no), tener un trabajo, empleo o empresa (sí o no), clasificación económica (A; B; C/D/E), padres que viven juntos (sí o no), tipo de colegio (público o privado); religión (ninguna; católica; evangélica; otra).

### Procesamiento y análisis de datos

El análisis estadístico se realizó inicialmente describiendo los datos mediante la presentación de frecuencias absolutas y relativas e intervalos de confianza del 95% (IC 95%), y se consideró la exclusión por caso completo, seguido de un análisis bivariante utilizando la prueba de Chi-cuadrado de Pearson (χ²) y la prueba exacta de Fisher, para verificar las posibles relaciones entre las variables. Posteriormente, se realizó un análisis multivariante bruto y ajustado de la Razón de Prevalencia (RP) mediante regresión de Poisson con varianza robusta, y se consideraron variables con significación estadística de 0,20 para su entrada en el ajuste, manteniendo aquellas con un valor p<0,05. Todos los análisis se realizaron utilizando el programa estadístico Stata 17.0.

### Aspectos éticos

El proyecto fue presentado al Comité de Ética de la Investigación de la Universidad Federal de Espírito Santo y aprobado por el dictamen núm. 5.900.370, para cumplir con la Resolución n.º 466/2012. Y obtuvo la firma de los responsables del Formulario de Consentimiento Informado (FCI) y de los adolescentes del Formulario de Asentimiento Libre e Informado (TALE). 

## Resultados

La prevalencia del uso constante de un estimulante a lo largo de su vida entre los escolares fue del 6% (IC 95%: 5,4-6,8), y actualmente el 1,6% utiliza alguna droga estimulante (IC 95%: 1,3-2,0). La prevalencia de escolares que informaron haber consumido cocaína al menos una vez en su vida fue del 1,2% (IC 95%: 1,0-1,6), y el consumo actual de esta droga es del 0,2% (IC 95%: 0,1-0,4). En cuanto al uso de crack o merla, la prevalencia fue del 0,1% (N=6, IC 95%: 0,0-0,2), y el uso actual fue del 0,1% (IC 95%: 0,0-0,2). Aproximadamente el 5% de la muestra afirmó haber experimentado éxtasis, bala o MD a lo largo de su vida, y actualmente el 1,4% usa esta droga (IC 95%: 1,1-1,8). En cuanto al consumo de metanfetamina, el 0,6% de los entrevistados afirmó haber probado ya metanfetamina en su vida (IC 95%: 0,4-0,9) y el 0,1% utiliza metanfetamina actualmente (IC 95%: 0,0-0,3) (Tabla 1).

**Tabla 1:** Uso de drogas estimulantes entre estudiantes de educación secundaria. Región Metropolitana de la Gran Vitória, ES, Brasil, marzo a diciembre, 2023

	N*	%^†^	IC95%^‡^
Uso alguna vez en la vida			
Drogas estimulantes			
No	4334	94,0	93,2-94,6
Sí	278	6,0	5,4-6,8
**Cocaína**			
No	4555	98,8	98,4-99,0
Sí	57	1,2	0,9-1,6
**Crack o merla**			
No	4604	99,9	99,7-99,9
Sí	06	0,1	0,01-0,3
**Éxtasis**			
No	4383	95,1	94,4-95,7
Sí	227	4,9	4,3-5,6
**Metanfetamina**			
No	4581	99,4	99,1-99,6
Sí	29	0,6	0,4-0,9
**Uso actualmente**			
**Drogas estimulantes**			
No	4538	98,4	97,9-98,7
Sí	74	1,6	1,3-2,0
**Cocaína**			
No	4602	99,8	99,6-99,9
Sí	10	0,2	0,1-0,4
**Crack o merla**			
No	4607	99,9	99,8-100,0
Sí	3	0,1	0,0-0,2
**Éxtasis**			
No	4544	98,6	98,2-98,9
Sí	66	1,4	1,1-1,8
**Metanfetamina**			
No	4603	99,9	99,7-99,9
Sí	7	0,1	0,0-0,3

*N = Frecuencia absoluta; ^†^% = Prevalencia; ^‡^IC95% = Intervalo de confianza del 95%

Según la Tabla 2, la edad a la que se usaron drogas estimulantes por primera vez fue de 15 años o más para todas las sustancias analizadas, y la proporción de inicios de consumo a partir de esa edad fue del 50% para cocaína (IC 95%: 36,7-63,3), 50% para crack (IC 95%: 10,9-89,1), 77,9% para éxtasis (IC 95%: 72,0-82,8) y 64% para consumo de metanfetamina (IC 95%: 42,9-80,8). Además, según los participantes, el 32,1% obtuvo cocaína de un amigo (IC 95%: 21,1-45,7), y, de igual manera, el éxtasis se adquirió a través de amigos para aproximadamente el 39% de los adolescentes (IC 95%: 33,0-45,7). Por otro lado, el crack/merla tenía una mayor frecuencia de compra en tiendas, bares, bares, con un vendedor y/o en el club (50%; IC 95%: 10,9-89,1), y, finalmente, aproximadamente el 28% de los adolescentes compraron y/o se dieron a discotecas (13,8%, IC 95%: 5,0-32,5) o consumieron metanfetamina con amigos (IC 95%, IC 95%: 5,0-32,5). Cabe destacar la alta prevalencia de adolescentes que reportan no recordar cómo se adquirió la sustancia para todas las drogas.

**Tabla 2:** Uso de drogas estimulantes entre estudiantes de educación secundaria. Región Metropolitana de la Gran Vitória, ES, Brasil, marzo a diciembre, 2023

	Cocaína			Crack/merla			*Éx*tasis/bala/MD			Metanfetamina		
	N*	%^†^	IC95%^‡^	N*	%^†^	IC95%^‡^	N*	%^†^	IC95%^‡^	N*	%^†^	IC95%^‡^
¿Cuántos años tenías cuando lo probaste por primera vez?												
Menos de 10 años	10	18,5	10,1-31,5	2	33,3	5,1-82,2	7	3,1	1,5-6,4	3	12,0	3,7-32,7
11 a 14 años	17	30,5	20,3-45,3	1	16,7	1,2-77,0	43	19,0	14,4-24,7	6	24,0	10,7-45,4
15 o más	27	50,0	36,7-63,3	3	50,0	10,9-89,1	176	77,9	72,0-82,8	16	64,0	42,9-80,8
**¿Cómo la obtuviste?**												
Lo compré en una tienda, bar, taberna, con un vendedor y/o en el club (fiesta)	9	16,1	8,5-28,4	3	50,0	10,9-89,1	54	23,8	18,7-29,8	4	13,8	5,0-32,5
Le di dinero a alguien para que me comprara	4	7,1	2,6-17,9	1	16,7*	1,2-77,0	9	4,0	2,1-7,5	-	-	-
Lo pillé escondido en casa	3	5,4	1,7-15,7	-	-	-	-	-	-	2	6,9	1,2-25,0
Una persona mayor me lo dio	1	1,8	2,4-12,1	-	-	-	21	9,3	6,1-13,8	-	-	-
Alguien de mi familia me lo dio	1	1,8	2,4-12,1	1	16,7*	1,2-77,0	4	1,8	0,7-4,2	3	10,3	3,2-28,7
Lo conseguí con un amigo	18	32,1	21,1-45,7	-	-	-	89	39,2	33,0-45,7	4	13,8	5,0-32,5
Lo conseguí en la escuela	-	-	-	-	-	-	-	-	-	1	3,5	0,4-22,3
No lo recuerdo y otra forma	20	35,7	24,1-49,3	1	16,7*	14,3	50	22,0	17,1-27,9	15	51,7	33,4-69,6

Nota: no hay posibilidad de redondear, porcentaje por encima; *N = Frecuencia absoluta; **†**% = Prevalencia; **‡**IC95% = Intervalo de confianza del 95%

La Tabla 3 muestra la distribución del consumo de drogas estimulantes entre los estudiantes de secundaria, según las características en estudio. Se observa una relación entre la experimentación y el uso de estimulantes y las variables: identidad de género, orientación sexual, raza/color de piel, estado civil, tipo de escuela, trabajo actual y religión (p<0.05). Por otro lado, las variables de edad y estado civil de los padres solo mostraron una relación significativa con la experimentación con estimulantes (p<0,001) y el uso actual de la droga según el género y la clase económica (p<0,05).

**Tabla 3:** Distribución del consumo de drogas estimulantes entre los estudiantes de educación secundaria de la Región Metropolitana de Gran Vitória. ES, Brasil, marzo a diciembre, 2023

Variables	Experimentación con estimulantes				Uso actual de estimulantes			
	N*	%^†^	IC95%^‡^	valor p	N*	%^†^	IC95%^‡^	valor p
**>Sexo^§^**				0,137				0,009
Mujeres	139	5,5	4,72-6,52		29	1,2	0,81-1,66	
Hombres	139	6,6	5,61-7,74		45	2,1	1,60-2,84	
**>Edad (años)**				<0,001				0,248
14 a 15	37	3,8	2,3-5,2		12	1,2	0,70-2,27	
16 a 17	179	5,9	5,1-6,8		48	1,6	1,29-2,09	
18 a 19	62	10,3	8,0-13,0		14	2,3	1,38-3,88	
**>Identidad de género**				0,032				0,010
Cisgénero	254	5,8	5,18-6,58		64	1,5	1,16-1,89	
No cisgénero	26	8,9	6,15-12,8		10	3,4	1,86-6,27	
**>Orientación sexual**				<0,001				0,049
Heterosexual	193	5,3	4,59-6,04		52	1,4	1,10-1,86	
LGBTQIA+	85	9,0	7,31-11,00		22	2,3	1,53-3,50	
**>Raza/Color de la piel**				0,001				0,003*
Blanca(o)	83	4,7	3,8-5,8		16	0,9	0,60-1,48	
Negra(o)	66	8,7	6,9-10,9		21	2,8	1,81-4,20	
Parda(o)	113	5,8	4,8-6,3		30	1,5	1,08-2,19	
**>Estado civil**				0,001				0,006
Con la pareja	98	8,0	6,59-9,63		44	1,3	0,97-1,74	
Sin compañero	180	5,3	4,61-6,12		30	2,4	1,71-3,47	
**>Clasificación socioeconómica**				0,957				0,017*
A	497	6,2	4,89-8,40		10	1,3	0,71-2,42	
B	129	5,9	5,05-7,05		47	2,2	1,62-2,86	
C//D/E	102	6,1	5,12-7,44		17	1,0	0,63-1,63	
**>Tipo de escuela**				<0,001				0,019
Pública	217	7,0	6,17-8,00		59	1,9	1,48-2,46	
Privada	61	4,0	3,13-5,12		15	1,0	0,59-1,63	
**>Actualmente, tiene un trabajo, empleo o negocios**				<0,001				<0,001
No	176	5,04	4,36-5,81		40	1,1	0,84-1,56	
Sí	102	9,12	7,58-11,00		31	3,0	2,18-4,23	
**>Religión**				<0,001				<0,001
Ninguna	91	8,4	6,85-10,15		25	2,3	1,56-3,38	
Católica	55	4,7	3,60-6,33		11	0,9	0,52-1,68	
Evangélica	89	4,4	3,62-4,43		22	1,1	0,72-1,66	
Otras	43	12,7	9,57-16,72		16	4,7	2,92-7,59	
**>Estado civil de los padres - viven juntos**				<0,001				0,056
Sí	99	4,10	3,37-4,96		29	1,2	0,83-1,72	
No	174	8,04	7,00-9,26		41	1,9	1,40-2,56	

*N = Frecuencia absoluta; ^†^% = Prevalencia; ^‡^IC95% = Intervalo de confianza de 95%; ^§^Exacta de Fisher

Tras ajustar por factores de confusión, se observó que las personas de 18 a 19 años tenían una prevalencia 2,24 veces mayor (IC 95%: 1,47-3,40) de experimentación con drogas estimulantes en comparación con adolescentes de 14 a 15 años. Los escolares que se identificaban como LGBTQIA+ tenían una prevalencia de experimentación un 35% mayor (IC 95%: 1,04-1,77) en comparación con los heterosexuales, así como los estudiantes negros tenían una prevalencia 1,55 veces mayor de experimentación con drogas estimulantes (IC 95%: 1,13-2,14) en comparación con los adolescentes blancos. La experimentación con estimulantes en algún momento de su vida fue un 68% más frecuente entre los estudiantes con trabajo (IC 95%: 1,32-2,13). Pertenencia a la religión católica redujo la prevalencia de la experimentación con drogas estimulantes en un 30% (PR: 0,70; IC 95% 0,50-0,99), así como en la religión evangélica, con una reducción del 41% (PR: 0,59; IC 95%: 0,44-0,81) en comparación con adolescentes que declararon no tener religión. Finalmente, los adolescentes cuyos padres viven separados tuvieron una frecuencia un 62% mayor de experimentación con drogas estimulantes (IC 95%: 1,26-2,10) en comparación con aquellos cuyos padres viven juntos (Tabla 4).

**Tabla 4:** Índice de prevalencia bruto y ajustado de la experimentación con drogas estimulantes entre estudiantes de educación secundaria. Región Metropolitana de la Gran Vitória, ES, Brasil, marzo a diciembre, 2023

Variables	Experimentación con drogas estimulantes					
	RP bruta*	IC95%^†^	valor p	RP ajustada^‡^	IC95%^†^	valor p
**Sexo**			0,138			0,155
Mujeres	1,0			1,0		
Hombres	1,19	0,95-1,49		1,20	0,93-1,53	
**Edad (años)**			<0,001			<0,001
14 a 15	1,0			1,0		
16 a 17	1,55	1,09-2,19		1,48	1,03-2,13	
18 a 19	2,69	1,81-3,99		2,24	1,47-3,40	
**Identidad de género**			0,031			0,862
Cisgénero	1,0			1,0		
No cisgénero	1,53	1,04-2,25		0,96	0,61-1,51	
**Orientación sexual**			<0,001			0,027
Heterosexual	1,0			1,0		
LGBTQIA+	1,70	1,33-2,17		1,35	1,04-1,77	
**Raza-color de la piel**			<0,001			0,025
Blanca(o)	1,0			1,0		
Negra(o)	1,86	1,36-2,54		1,55	1,13-2,14	
Parda(o)	1,24	0,94-1,63		1,19	0,90-1,58	
**Estado civil**			0,001			0,063
Con la pareja	1,50	1,18-1,90		1,27	0,99-1,63	
Sin compañero	1,0			1,0		
**Clasificación socioeconómica**			0,957			-
A	1,01			-	-	
B	0,98	0,75-1,25		-	-	
C//D/E	1,0	0,72-1,41		-	-	
**Tipo de escuela**			<0,001			0,177
Pública	1,75	1,33-3,31		1,24	0,91-1,68	
Privada	1,0			1,0		
**Actualmente, tiene algún trabajo, empleo o negocios**			<0,001			<0,001
No	1,0			1,0		
Sí	1,81	1,43-2,29		1,68	1,32-2,13	
**Religión**			<0,001			<0,001
Ninguna	1,0			1,0		
Católica	0,56	0,40-0,77		0,70	0,50-0,99	
Evangélica	0,53	0,40-0,70		0,59	0,44-0,81	
Otras	1,52	1,08-2,14		1,36	0,94-1,95	
**Estado civil de los padres - viven juntos**			<0,001			<0,001
Sí	1,0			1,0		
No	1,96	1,53-2,51		1,62	1,26-2,10	

*RP bruta = Razón de prevalencia bruta; †IC95% = Intervalo de confianza de 95%; ‡RP ajustada = Razón de prevalencia ajustada

El uso actual de drogas estimulantes tras ajustar los datos fue más prevalente en adolescentes negros (RP 2,94; IC 95%: 1,53-5,65) en comparación con adolescentes de raza blanca. En comparación con los adolescentes de categorías económicas inferiores (C/D/E), los adolescentes de clase B tenían una prevalencia 2,27 veces mayor en el uso actual de drogas estimulantes (IC 95%: 1,31-3,95). Tener un empleo llevó a una prevalencia 2,69 veces mayor del consumo actual (IC 95%: 1,69-4,30) en comparación con adolescentes sin trabajo, y la prevalencia del consumo actual de drogas estimulantes fue un 58% menor en quienes pertenecían a la religión católica (IC 95%: 0,20-0,87) y un 57% menor en los escolares evangélicos (IC 95%: 0,24-0,78), en comparación con los adolescentes que declararon no tener religión (Tabla 5).

**Tabla 5:** Análisis bruto y ajustado del consumo actual de drogas estimulantes entre estudiantes de educación secundaria. Región Metropolitana de la Gran Vitória, ES, Brasil, marzo a diciembre, 2023

Variables	Uso actual de drogas estimulantes					
	RP bruta*	IC95%^†^	valor p	RP ajustada^‡^	IC95%^†^	valor p
**Sexo**			0,010			0,103
Mujeres	1,0			1,0		
Hombres	1,84	1,16-2,93		1,49	0,92-2,41	
**Edad (años)**			0,254			-
14 a 15	1,0			-	-	
16 a 17	1,28	0,68-2,40		-	-	
18 a 19	1,87	0,87-4,02		-	-	
**Identidad de género**			0,012			0,445
Cisgénero	1,0			1,0		
No cisgénero	2,32	1,20-4,47		1,39	0,59-3,28	
**Orientación sexual**			0,050			0,223
Heterosexual	1,0			1,0		
No Heterosexual	1,63	1,00-2,68		1,45	0,80-2,64	
**Raza/Color de la piel**			0,003			0,005
Blanca(o)	1,0			1,0		
Negra(o)	3,06	1,61-5,84		2,94		
Parda(o)	1,71	0,93-3,12		1,74	1,53-5,65	
**Estado civil**			0,007		0,97-3,12	0,098
Sin pareja	1,0			1,0		
Con compañero	1,89	1,19-2,97		1,52	0,93-2,49	
**Clasificación socioeconómica**			0,019			0,009
A	2,12	0,59-2,79		1,26	0,52-3,03	
B	1,29	1,22-3,67		2,27	1,31-3,95	
C//D/E	1,0			1,0		
**Tipo de escuela**			0,021			0,672
Pública	1,94	1,10-3,40		1,14	0,61-2,13	
Privada	1,0			1,0		
**Actualmente, tiene algún trabajo, empleo o negocios**			<0,001			<0,001
No	1,0			1,0		
Sí	2,66	1,69-4,18		2,69	1,69-4,30	
**Religión**			<0,001			<0,001
Ninguna	1,0			1,0		
Católica	0,41	0,20-0,82		0,42	0,20-0,87	
Evangélica	0,48	0,27-0,84		0.43	0,24-0,78	
Espiritista	2,06	1,11-3,82		1,49	0,75-2,94	
**Estado civil de los padres - viven juntos**			0,060			0,395
Sí	1,0			1,0		
No	1,58	0,98-2,54		1,24	0,76-2,03	

*RP bruta = Razón de prevalencia bruta; ^†^IC95% = Intervalo de confianza del 95%; ^‡^RP ajustada = Razón de prevalencia ajustada

## Discusión

La prevalencia del consumo de drogas estimulantes al menos una vez en la vida entre los escolares de este estudio fue del 6% (IC 95%: 5,4-6,8), mientras que el consumo actual fue del 1,6% (IC 95%: 1,3-2,0). Estos hallazgos están en línea con los datos publicados por el estudio norteamericano *Monitoring the Future* (MTF, 2022), que investigó a adolescentes de educación secundaria hasta 18 años, identificando una prevalencia similar de consumo al menos una vez en la vida (6,1%) y uso actual (1,5%) de drogas estimulantes. En el mismo sentido, un estudio realizado en Chile con adolescentes de 13 a 17 años también observó una prevalencia del 5,9% del uso de estimulantes al menos una vez en la vida y del 1,7% del consumo actual^18^.

En cuanto al consumo de cocaína, se observó que la prevalencia de experimentación en la vida entre los escolares fue del 1,2% (IC 95%: 1,0-1,6). Este resultado está en línea con un estudio realizado en Nigeria, en el que el 1,4% de los adolescentes declaró haber usado esta sustancia a lo largo de su vida^19^. De manera similar, un estudio realizado en Macao, China, identificó una prevalencia del 1,2% del consumo de cocaína al menos una vez en la vida entre adolescentes^20^. Por otro lado, un estudio realizado en Brasil, en la ciudad de Cuiabá, encontró una mayor prevalencia de consumo de cocaína a lo largo de la vida (3,1%)^21^. En cuanto al consumo actual de cocaína, se observó una prevalencia del 0,2% (IC 95%: 0,1-0,4). Este resultado es similar al descrito en estudios con la población adolescente, en los que el 0,4% de los adolescentes reportó un consumo actual de cocaína^19^.

En cuanto al consumo de crack o merla, la prevalencia encontrada fue del 0,1% (IC 95%: 0,0-0,2). Este resultado es inferior al identificado por el Proyecto de Investigación de la Escuela Europea sobre Alcohol y Otras Drogas, que analizó datos de 99.647 adolescentes en 35 países y encontró una prevalencia media del 1,1% de consumo de crack al menos una vez en la vida. En este estudio, la prevalencia más alta se observó en Chipre (3,1%), mientras que la más baja se dio en Macedonia del Norte (0,2%) y Eslovaquia (0,5%)^9^. De manera similar, un estudio transversal realizado en Brasil, en la ciudad de Cuiabá (MT), identificó una prevalencia del 1,4% del uso de crack al menos una vez en la vida entre adolescentes^21^.

En cuanto al consumo de éxtasis, bala o MD, la prevalencia en algún momento de la vida fue del 5%, similar a la identificada en el estudio *European School Survey Project on Alcohol and Other Drugs* (ESPAD)^9^, que reveló una prevalencia del 5,1% entre adolescentes de educación secundaria en los Países Bajos. De manera similar, la prevalencia de uso actual observada (1,4%; IC 95%: 1,1-1,8) está en línea con lo registrado en ESPAD^9^, pero con un enfoque en adolescentes en la República Checa, donde el 1,7% reportó el consumo de estas drogas.

La prevalencia del consumo de metanfetamina al menos una vez en la vida entre los escolares fue del 0,6% (IC 95%: 0,4-0,9), un valor más alto, aunque no muy lejos, que el observado en el estudio del *Centro Brasileiro de Informações sobre Drogas Psicotrópicas* (CEBRID, 2020), que identificó una prevalencia inferior al 0,1% para el uso de esta sustancia. Además, el consumo actual de metanfetamina en este estudio fue del 0,1% (IC 95%: 0,0-0,3), lo que indica un bajo consumo entre los escolares, un resultado similar al descrito por ESPAD (2019), que indicaba una prevalencia del 0,3% entre los adolescentes^9^.

El análisis de la edad en la que comienza el consumo de drogas estimulantes revela una tendencia preocupante: para todas las sustancias analizadas, la primera experiencia ocurre a partir de los 15 años. Alrededor del 27,9% de las personas comienzan a usar drogas estimulantes antes de los 18 años, con una tendencia más pronunciada entre los hombres, que puede reflejar diferencias sociales, culturales y biológicas en la propensión al consumo^15^. El inicio temprano del uso de estas sustancias está estrechamente asociado con el desarrollo de problemas más graves relacionados con el abuso y la dependencia de drogas en la edad adulta^16^.

Los datos de la encuesta indican que la adquisición de estimulantes se realiza a través de amigos o comprando en tiendas, bares, con un vendedor y/o en el club. Los estudios han reportado una mayor frecuencia de jóvenes que adquieren drogas estimulantes (éxtasis, anfetaminas, cocaína) en bares, discotecas, fiestas, etc.^22^ ^-^ ^25^. Esto es probable que ocurra porque fiestas como *raves* tienen más probabilidades de estar asociadas con mayores probabilidades de consumir estas drogas, ya que en estas fiestas se usan drogas para mejorar la música, la luz, la socialización y el baile durante toda la noche^22^. 

Otro hallazgo fue la mayor prevalencia de experimentación con estimulantes entre adolescentes mayores de 18 a 19 años (IC 95%: 1,47-3,40). Además de los hallazgos del presente estudio, se identificó que el uso de este tipo de drogas lo realizan jóvenes mayores^14^. Además, hay informes en la literatura de una edad media de 18-19 años para el *cannabis* y de 21-24 años para la cocaína^26^. Además, entre 2000 y 2001, ya existía evidencia de un gran aumento en el uso de estimulantes tipo anfetaminas entre adolescentes en Asia y Europa^27^. 

Además, hubo una mayor experimentación con estimulantes entre jóvenes que se declararon LGBTQIA+, con un 35% más de prevalencia (IC 95%: 1,04-1,77). Estos datos son similares a los de otros estudios, que sugieren que este grupo poblacional tiene un mayor riesgo de usar estas sustancias relacionadas con factores de estrés minoritarios, como el estigma sufrido^28^ ^-^ ^29^.

Además, en nuestro estudio, hay una mayor frecuencia de experimentación y uso actual de estimulantes entre personas negras, datos que aún no se han explorado en la literatura. Un estudio realizado en Estados Unidos muestra que los jóvenes de piel blanca tienen más probabilidades de recibir tratamiento y de acceso a servicios de salud privados^30^. 

El presente estudio encontró una mayor prevalencia de experimentación y uso actual de estimulantes entre estudiantes trabajadores, datos en línea con un estudio que utilizó datos de más de 109 mil estudiantes brasileños y encontró una mayor frecuencia de consumo de drogas entre adolescentes trabajadores, y sugiere que el trabajo remunerado puede permitir una mayor independencia financiera e influir en la autonomía de los adolescentes en la toma de decisiones sobre el consumo de drogas^31^.

La asociación entre el uso de estimulantes y marcadores sociales como la raza/color y la pertenencia a grupos socialmente estigmatizados, como las personas LGBTQIA+, pone de manifiesto el papel de las desigualdades estructurales en la producción de vulnerabilidades en la salud. El racismo y el prejuicio estructurales actúan como determinantes sociales que aumentan la exposición a factores de estrés crónicos, debilitan las redes de apoyo y restringen el acceso oportuno a servicios de salud y protección social, especialmente durante la adolescencia^32^. En este contexto, la teoría del estrés minoritario es un enfoque interseccional, según el cual la superposición de múltiples marcadores minoritarios intensifica la exposición a factores de riesgo y potencia los impactos negativos en la salud mental. Sostiene que las experiencias persistentes de estigmatización, discriminación y violencia aumentan el riesgo de sufrimiento psicológico y consumo de sustancias como estrategia de afrontamiento^33^.

Pertenecer a la religión católica o evangélica disminuyó la prevalencia de la experimentación y el uso actual de drogas estimulantes en comparación con los adolescentes que declaraban no tener religión. Los hallazgos corroboran un estudio transversal realizado en el interior de São Paulo, en el que los estudiantes que practicaban una religión tenían menos probabilidades de consumir drogas^34^. La religiosidad despierta una mezcla de miedo y tabú y, en forma de creencias y prácticas, puede verse como un factor fuerte de resiliencia y resistencia contra el consumo de sustancias por parte de los adolescentes^35^.

Otro hallazgo fue el uso actual más frecuente en adolescentes que pertenecen a clasificaciones económicas más altas. Una encuesta transversal realizada en Nigeria a más de 1.000 adolescentes en escuelas públicas y privadas encontró que los estudiantes que asisten a escuelas en zonas pobladas por personas con mayores ingresos tienen más probabilidades de abusar de drogas, incluida la cocaína. Es posible que exista una relación con el mayor poder adquisitivo disponible para los estudiantes en esas áreas^19^. 

También se encontró que los adolescentes cuyos padres viven separados tenían un 62% más de frecuencia de experimentación con drogas estimulantes (IC 95%: 1,26-2,10) en comparación con aquellos cuyos padres viven juntos. Este resultado está en línea con un estudio realizado en Estados Unidos con datos de más de 41.000 adolescentes, en el que quienes pertenecían a familias monoparentales y no parentales tenían más probabilidades de consumir cocaína/heroína/metanfetamina (OR = 1,63-1,78, IC 95% = 1,21-2,71) que los jóvenes en familias biparentales^36^. Una de las posibles justificaciones sería que las familias con dos progenitores son más propensas a vigilar de cerca a los jóvenes, por lo que serían menos propensos a estar expuestos a conductas de riesgo^36^. Además, además de la relación entre los padres, el tiempo dedicado a sus hijos puede influir en el consumo de sustancias psicoactivas^37^. 

Teniendo en cuenta los resultados presentados y los impactos del uso de sustancias psicoactivas en la salud, los profesionales de enfermería desempeñan un papel importante en este escenario. La atención de enfermería para adolescentes que consumen drogas o tienen alto riesgo de consumirlas debe basarse en una actitud acogedora, sin prejuicios y capaz de proporcionar un espacio para el diálogo. Se deben utilizar estrategias de promoción y prevención de la salud, guiadas por la mejor evidencia científica, centradas en la autonomía y la reducción de daños del sujeto^38^. Las acciones pueden llevarse a cabo en la propia consulta individual de enfermería o en actividades colectivas. De este modo, es posible esbozar estrategias para que sean más reflexivos, competentes y seguros de sí mismos. Cuando se estimulan las potencialidades, se fortalece la autoestima para permitir elecciones y decisiones conscientes^39^.

El diseño transversal tiene limitaciones en cuanto a la determinación de la causalidad, ya que el resultado se evalúa al mismo tiempo que la exposición, lo que impide la identificación de la temporalidad de los eventos, requiriendo precaución al evaluar las relaciones entre las variables exploradas. Además, puede producirse sesgo informativo que compromete la precisión de las respuestas obtenidas; sin embargo, conviene considerar que los estudiantes pudieron responder el cuestionario de forma individual, respetando su privacidad y reduciendo así el sesgo mencionado anteriormente.

## Conclusión

Podemos concluir que el 6% de los adolescentes de secundaria ya han consumido drogas estimulantes en algún momento de sus vidas, y que el 1,6% las usa actualmente. La edad de uso, para la mayoría, fue de 15 años o más, y la adquisición de esta sustancia ocurre principalmente en tiendas, bares, bares, con un vendedor y/o en el club. Se observó una mayor prevalencia de experimentación entre adolescentes mayores (de 18 a 19 años) que se autodeclararon LGBTQIA+, negros, escolares con trabajo y cuyos padres viven por separado. El uso actual es más frecuente entre quienes pertenecen a la clase B, de raza negra y que trabajan. Quienes tienen una religión (evangélica o católica) tenían menor prevalencia de experimentación y uso actual.

Los hallazgos del presente estudio refuerzan la necesidad de estrategias de afrontamiento frente al uso de drogas estimulantes entre adolescentes en contextos comunitarios, familiares y escolares. Los esfuerzos para coordinar la salud, la educación, la asistencia social y la seguridad pública intersectoriales, así como para incluir a la familia en este proceso, deben emplearse para prevenir y mitigar el uso. En el ámbito académico, es esencial realizar estudios con un enfoque cualitativo que exploren, principalmente, las razones relacionadas con el mayor consumo entre estos adolescentes, además del desarrollo de investigaciones sobre intervenciones basadas en fortalecer las acciones preventivas. Por último, es necesario potenciar los factores que reducen la exposición al riesgo de usar estas sustancias para promover una juventud saludable.

## Data Availability

Los conjuntos de datos relacionados con este artículo estarán disponibles previa solicitud al autor correspondiente.
